# Single and Multiple Dose Pharmacokinetics, Pharmacodynamics and Safety of the Novel Lipoprotein-Associated Phospholipase A_2_ Enzyme Inhibitor Darapladib in Healthy Chinese Subjects: An Open Label Phase-1 Clinical Trial

**DOI:** 10.1371/journal.pone.0139862

**Published:** 2015-10-14

**Authors:** Chaoying Hu, Debra Tompson, Mindy Magee, Qian Chen, Yan Mei Liu, Wenjing Zhu, Hongxin Zhao, Annette S. Gross, Yun Liu

**Affiliations:** 1 Phase I Clinical Research Unit, Shanghai Xuhui Central Hospital, Shanghai, China; 2 Clinical Pharmacology Modeling and Simulation, GSK Medicines Research Centre, Stevenage, United Kingdom; 3 Clinical Pharmacology Modeling and Simulation, GlaxoSmithKline, King of Prussia, Pennsylvania, United States of America; 4 China Medicine Development, GlaxoSmithKline (China) R&D Company Limited, Shanghai, China; 5 Ethnopharmacology, GlaxoSmithKline, Sydney, Australia; National Cancer Centre, SINGAPORE

## Abstract

**Background and Objectives:**

Darapladib is a lipoprotein-associated phospholipase A_2_ (Lp-PLA_2_) inhibitor. This study evaluated the pharmacokinetics, pharmacodynamics and safety of darapladib in healthy Chinese subjects.

**Methods:**

Twenty-four subjects received darapladib 160 mg orally, approximately 1 hour after a standard breakfast, as a single dose and once daily for 28 days. Non-compartmental methods were used to determine the single and multiple dose pharmacokinetics of darapladib and its metabolite SB-553253. Repeat dose Lp-PLA_2_ activity and safety were evaluated.

**Results:**

Systemic exposure (AUC_(0-T)_, Cmax geometric mean (CVb%)) of darapladib was higher after multiple-dosing (519 ng.h/mL (33.3%), 34.4 ng/mL (49.9%)) compared to single-dose administration (153 ng.h/mL (69.0%), 17.9 ng/mL (55.2%). The steady-state accumulation ratio was less than unity (Rs = 0.80), indicating time-dependent pharmacokinetics of darapladib. Darapladib steady-state was reached by Day 14 of once daily dosing. Systemic exposure to SB-553253 was lower than darapladib with median (SB-553253: darapladib) ratios for AUC_(0-τ)_ of 0.0786 for single dose and 0.0532 for multiple dose administration. On Day 28, pre-dose and maximum inhibition of Lp-PLA_2_ activity was approximately 70% and 75% relative to the baseline value, respectively and was dependent of darapladib concentration. The most common adverse events (≥ 21% subjects) were abnormal faeces, abnormal urine odour, diarrhoea and nasopharyngitis.

**Conclusion:**

Darapladib 160 mg single and repeat doses were profiled in healthy Chinese subjects. Single dose systemic exposure to darapladib in healthy Chinese subjects was consistent with that observed previously in Western subjects whereas steady-state systemic exposure was approximately 65% higher in Chinese than Western subjects. The Lp-PLA_2_ activity and adverse event profile were similar in healthy Chinese and previous reports in Western subjects. Ethnic-specific dose adjustment of darapladib is not considered necessary for the Chinese population.

**Trial Registration:**

ClinicalTrials.gov NCT02000804

## Introduction

Lipoprotein-associated phospholipase A_2_ (Lp-PLA_2_), an enzyme produced and secreted by inflammatory cells, has the potential to be a novel target for the treatment of atherosclerosis [1–3] among other indications. Darapladib is a first-in-class orally active, selective, reversible inhibitor of the Lp-PLA_2_ enzyme that is currently being developed for the treatment of atherosclerosis [4] and diabetic macular edema [5]. Darapladib is administered as an enteric coated (EC) tablet containing the micronized free base of darapladib. According to the biopharmaceutical classification system (BCS), darapladib is considered to be a Class 4 substance due to its low passive permeability (≤7 nm/s at pH 5.5) and low solubility (>1, 0.8, 0.3 and <0.1 mg/mL at pH 2, 4, 6 and 8, respectively) [Unpublished data]. Consistent with Class 4 BCS products, the absolute bioavailability of oral darapladib is low (4% for darapladib enteric coated tablet [Unpublished data] and 12% for a solution [6]). Compared with the fasted state, administration of a high fat meal with darapladib enteric coated tablet resulted in a 19% increase in systemic exposure (AUC) to darapladib and the higher exposure is not considered to be clinically relevant [Unpublished data].

Following oral absorption, the principal circulating drug-related component is unchanged darapladib (75%) [6]. Circulating concentrations of the darapladib metabolites, SB-823094, SB-553253 and SB-554008, are low with each accounting for ≤5% of total drug related material [6]. The pharmacological activities of SB-823094 and SB-553253 are similar to that of darapladib, while of the activity of SB-554008 is about a 100 times less than the parent drug. The low exposures of darapladib metabolites relative to the parent drug suggest that metabolites do not contribute significantly to the inhibition of Lp-PLA_2_ observed in humans. *In vitro* studies demonstrate that the metabolism of darapladib is mediated primarily by cytochrome P450 (CYP) 3A4 with minor contributions from other CYPs, most notably CYP2C8 [6].

Darapladib exhibits time-dependent pharmacokinetics. In Western subjects, the steady-state accumulation ratio, Rs (AUC_(0-τ), repeat dose_/ AUC _(0-∞), single dose_) was less than unity (0.56 [90% CI = 0.48, 0.66]) and the observed accumulation ratio (R_o_) following 28 days of once daily dosing was lower (R_o_ = 2.08) than predicted from single dose data (R_p_ = 3.70) [Unpublished data]. Based on the results of a drug-drug interaction study between darapladib and midazolam, there was no evidence that darapladib induces CYP3A4 metabolism [7]. The mechanism contributing to the change in darapladib pharmacokinetics on repeat dosing is consequently currently unclear.

The pharmacokinetics (PK), pharmacodynamics (PD) and safety of darapladib have been explored in both healthy Western and healthy Japanese subjects. There were no substantial inter-ethnic differences in the PK (darapladib and its metabolite SB-553253), PD (Lp-PLA_2_ activity) or safety between Japanese and Western subjects [Unpublished data].

The pharmacokinetics of drugs can differ between populations, reflecting differences in the intrinsic and extrinsic factors which influence the pharmacokinetics of a particular drug [8]. In order to support the potential registration of darapladib in the People’s Republic of China, this study was conducted to assess the pharmacokinetics of single and multiple once daily doses of 160 mg enteric coated darapladib in healthy Chinese subjects. Additionally, the repeat dose pharmacodynamics (effects on Lp-PLA_2_ activity) and safety of darapladib were assessed. The pharmacokinetics of metabolite SB-553253 was also characterized in the healthy Chinese subjects studied.

## Materials and Methods

### Study subjects

Healthy male or female Chinese subjects, aged 18–45 years (inclusive) with body weight ≥50 kg and body mass index within the range of 19–24 kg/m^2^ (inclusive) were eligible to participate in this study. Subjects were confirmed as healthy based on the review of their medical history, physical examination, vital signs and clinical laboratory tests. Women of child bearing potential had a negative urine pregnancy test and pregnant or lactating women were excluded. Subjects with a positive pre-study drug/alcohol screen, who were unable to refrain from the use of prescription or non-prescription drugs and subjects who had recently donated blood were also excluded. Subjects who consumed grapefruit or grapefruit juice within 7 days prior to first dose of study medication were excluded.

### Ethics statement

The study protocol, protocol amendments, and informed consent were reviewed and approved by Shanghai Xuhui Central Hospital Ethics Committee, Shanghai, China. This study was conducted in accordance with Good Clinical Practice and the guiding principles of the Declaration of Helsinki, and all subjects provided written informed consent prior to the performance of any study-specific procedures.

This study is registered at clinicaltrials.gov (NCT02000804).

### Study design

This Phase 1, open-label, single-arm study was conducted from October 2013 to January 2014 at a single center in China. Each subject enrolled in the study participated in 2 phases, a single dose phase and a multiple dose phase. The subjects received 160 mg enteric coated micronized free-base darapladib as a single dose in the single-dose phase and as once daily repeat doses for 28 days in the multiple-dose phase. On each dosing occasion, darapladib was administered approximately 1 hour after a standard breakfast. Blood samples for pharmacokinetic analysis were collected over a 96-hour period after single dose administration. There were at least 4 days between the single dose and the first dose of the multiple dose phase which allowed for the 96-hour sampling period following the single dose administered. On Day 28 of multiple dosing, blood samples for pharmacokinetic and pharmacodynamic analysis were collected over a 24-hour period after dosing. Subjects returned to the study centre approximately 28 days after the last dose of darapladib for a safety follow-up visit. The total study duration for each subject including the screening, treatment and follow-up periods was approximately 12 weeks.

### Pharmacokinetic assessments

Approximately 2 mL of whole blood was collected via an indwelling cannula (or by direct venepuncture) into tubes containing the anticoagulant K_2_ ethylenediaminetetraacetic acid (EDTA). In the single dose session, samples were collected at the following time points: predose (60 minutes before dosing), 0.5, 1, 2, 3, 4, 6, 9, 12, 15, 24, 36, 48, 72 and 96 hours after darapladib administration. In the multiple dose session, samples were collected on Day 28 of dosing at pre-dose and at 0.5, 1, 2, 3, 4, 6, 9, 12, 15, 24 hours post-dose. Sample collection was continued after the last multiple dose at the following time points: 36, 48, 72, 96, 120, 144, 168, 240, 312, 384, 456, 528, 600 and 672 hours. In addition, pre-dose (trough) blood samples were collected on Day 14, Day 26 and Day 27 to determine trough plasma concentrations (C_T_) of darapladib and SB-553253 and the approach to steady-state.

The blood samples were centrifuged within 60 mins at approximately 4°C at 1500g for 15 minutes and the supernatant plasma was stored at -20°C until further analysis. The plasma samples were analyzed for darapladib and SB-553253 using a validated analytical method at Wuxi AppTec Co. Ltd (Shanghai, China). The method was based on protein precipitation extraction, followed by liquid chromatography tandem mass spectrometry (LC-MS/MS) analysis. The lower limit of quantification (LLOQ) and the upper limit of quantification (ULOQ) were 0.100 to 50.0 ng/mL, respectively for darapladib and 0.250 to 50.0 ng/mL, respectively for SB-553253 using 50.0 μL of human plasma. The intra-assay precision and accuracy for darapladib were 1.3 to 5.0% and -3.7 to 7.1%, respectively, while inter-assay precision and accuracy were 2.1 to 4.6% and -1.3 to 4.0%, respectively. The intra-assay precision and accuracy for SB-553253 were 1.2 to 6.0% and -1.6 to 10.8%, respectively, whereas, inter-assay precision and accuracy were 2.4 to 6.6% and 0.9 to 4.4%, respectively. Standard non-compartmental pharmacokinetic parameters were calculated using WinNonlin version 5.3 [Pharsight Corporation, Mountain View, CA, USA].

The following pharmacokinetic parameters for single dose and multiple dose darapladib and SB-553253 were calculated from the plasma concentration-time data based on actual blood sampling times: maximum observed concentration (C_max_), the time of occurrence of C_max_ (T_max_) and the area under the concentration-time curve over the 24 hour dosing interval (AUC_[0-T]_). AUC was calculated using the linear and logarithmic trapezoidal methods, for increasing and decreasing concentrations, respectively. For single dose administration only, AUC from time zero (pre-dose) to infinite time AUC(0-∞) was calculated as AUC(0-t) + Ct λz, where t is the time of last quantifiable concentratin, Ct is the last quantifiable concentration and λz is the terminal plasma elimination rate-constant. Terminal phase half-life (t_1/2_) was calculated as ln2/λz. For single dose administration t½ was determined up to 96 hours post-dose whereas for multiple dose administration t½ was determined up to 672 hours post-dose. For the metabolite SB-553253 AUC _[0-T]_, C_max_, t_1/2_ and T_max_ were calculated.

To estimate the extent of accumulation after multiple dosing, the observed accumulation ratio (Ro), the predicted accumulation ratio (Rp), the steady-state accumulation ratio (Rs) and C_max_ accumulation ratio (RC_max_) for both darapladib and SB-553253 were determined using following formulae.

$$Ro=AUC_{\left(0-\gamma \right)},repeat\;dose/AUC_{\left(0-\gamma \right)},single\;dose$$

$$Rp=AUC_{\left(0-\infty \right)},single\;dose/AUC_{\left(0-\gamma \right)},single\;dose$$

$$Rs=AUC_{\left(0-\gamma \right)},repeat\;dose/AUC_{\left(0-\infty \right)},single\;dose$$

$$RC_{max=}C_{max,repeat\;dose}/C_{max},single\;dose$$

The attainment of steady-state was assessed based on visual inspection of the trough concentrations Cτ on Day 14, Day 26, Day 27 and Day 28 of the multiple dose phase.

The metabolite to parent ratios for AUC_[0-T]_ and C_max_ were calculated for SB-553253 relative to darapladib following single and multiple dosing.

### Pharmacodynamic assessments

Blood samples for the determination of Lp-PLA_2_ activity were collected on Day 28 following the last dose of the multiple dose phase at the same time points as for the pharmacokinetic sampling. In addition, pre-dose (trough) blood samples were collected on Days 14, Day 26 and Day 27. Plasma was separated by centrifugation (approximately 4°C) at approximately 1600 g for 15 minutes and stored in polypropylene tubes at approximately -20°C or cooler. The assay for Lp-PLA_2_ activity was performed using micro colorimetric activity method.

### Pharmacokinetic-pharmacodynamic relationship

The relationship between darapladib plasma concentration and plasma Lp-PLA_2_ activity (nmol/min/mL) was explored graphically. A sigmoid E_max_ model was used to describe the relationship between Lp-PLA_2_ activity and plasma concentrations of darapladib (C) based on the following equation.

$$Plasma\;Lp-PLA_{2}activity=E_{0}\times \left(1-\frac{C^{\gamma }}{C^{\gamma }+IC_{50}^{\gamma }}\right)$$

E_0_ is the baseline plasma Lp-PLA_2_ activity (nmol/min/mL), IC_50_ is the darapladib plasma concentration causing 50% inhibition of Lp-PLA_2_ activity and γ is the Hill coefficient which describes the steepness of the PK/PD relationship. The PK/PD data were analyzed using Nonlinear Mixed Effect Modeling as implemented in the computer program Nonlinear Mixed Effects Modeling (NONMEM) [Version 7.2, ICON Development Solutions, USA].

### Safety and tolerability evaluations

Safety data evaluations including assessment of treatment emergent adverse events (TEAEs), physical examination, clinical laboratory tests (hematology, clinical chemistry and urinalysis), electrocardiogram and vital sign measurements were performed throughout the study. The severity of adverse events was assessed by the investigator refer to the criteria of common terminology criteria for adverse events (CTCAE_4.03).

### Statistical analysis

The sample size was primary driven by feasibility and regulatory requirement. It was planned to recruit 24 subjects to have 18 evaluable subjects. With 18 subjects evaluable for pharmacokinetic analysis, the half width of the 90% confidence interval (CI) for RC_max_ would be within 22.0% of the point estimate and for R_o_, R_p_ and R_s_ would be within 16.6% of point estimate, assuming the within-subject coefficient of variation (CVw %) in this study would be 35.4% for C_max_ and 27.0% for AUC.

All subjects who received at least 1 dose of darapladib and provided evaluable pharmacokinetic data were included in the pharmacokinetic population. All subjects who received at least 1 dose of darapladib were included in the safety population. The subjects who provided data at baseline and with at least one post-dose observation to calculate percent inhibition of plasma Lp-PLA_2_ activity were included in the pharmacodynamic population.

SAS (version 9.2; SAS Institute Inc, Cary, NC, USA) was used for statistical analyses. The summary statistics of the pharmacokinetic parameters for darapladib and SB-553253 were calculated after log_e_ transformation. To evaluate accumulation ratio statistical analysis of log transformed AUC and C_max_ values was performed separately. A mixed effects model was fitted with day as a fixed effect and subject as a random effect to estimate R_o_, R_s_ and RC_max_. Point estimates and associated 90% CIs for the comparisons of interests were estimated. The point estimates and associated 90% CIs were then exponentially back-transformed to provide point estimates and 90% CIs for the ratios R_o_, R_s_ and R_Cmax_. A similar analysis was performed with single dose AUC_(0-∞)_ and single dose AUC_(0-T)_ data fitting PK parameter as fixed effect and subject as random effect to provide point estimates and 90% CIs for the ratio R_p_. The within subject coefficient of variation (CV_w_%) for AUC_(0-T)_ and C_max_ were calculated based on residual error. Pharmacodynamic activity was expressed in terms of percent inhibition of Lp-PLA_2_ enzyme activity relative to baseline.

The percent inhibition of plasma Lp-PLA_2_ activity at time x relative to baseline was calculated using the following formula:

$$100\;x\;\left[\left({\text{Activity}}_{\text{baseline }}{-\text{ Activity}}_{\text{time }x}\right){\;/\text{ Activity}}_{\text{baseline}}\right]$$

Where Activity represents plasma Lp-PLA_2_ activity.

Safety data were summarized descriptively.

## Results

### Demographics

**Fig 1:**
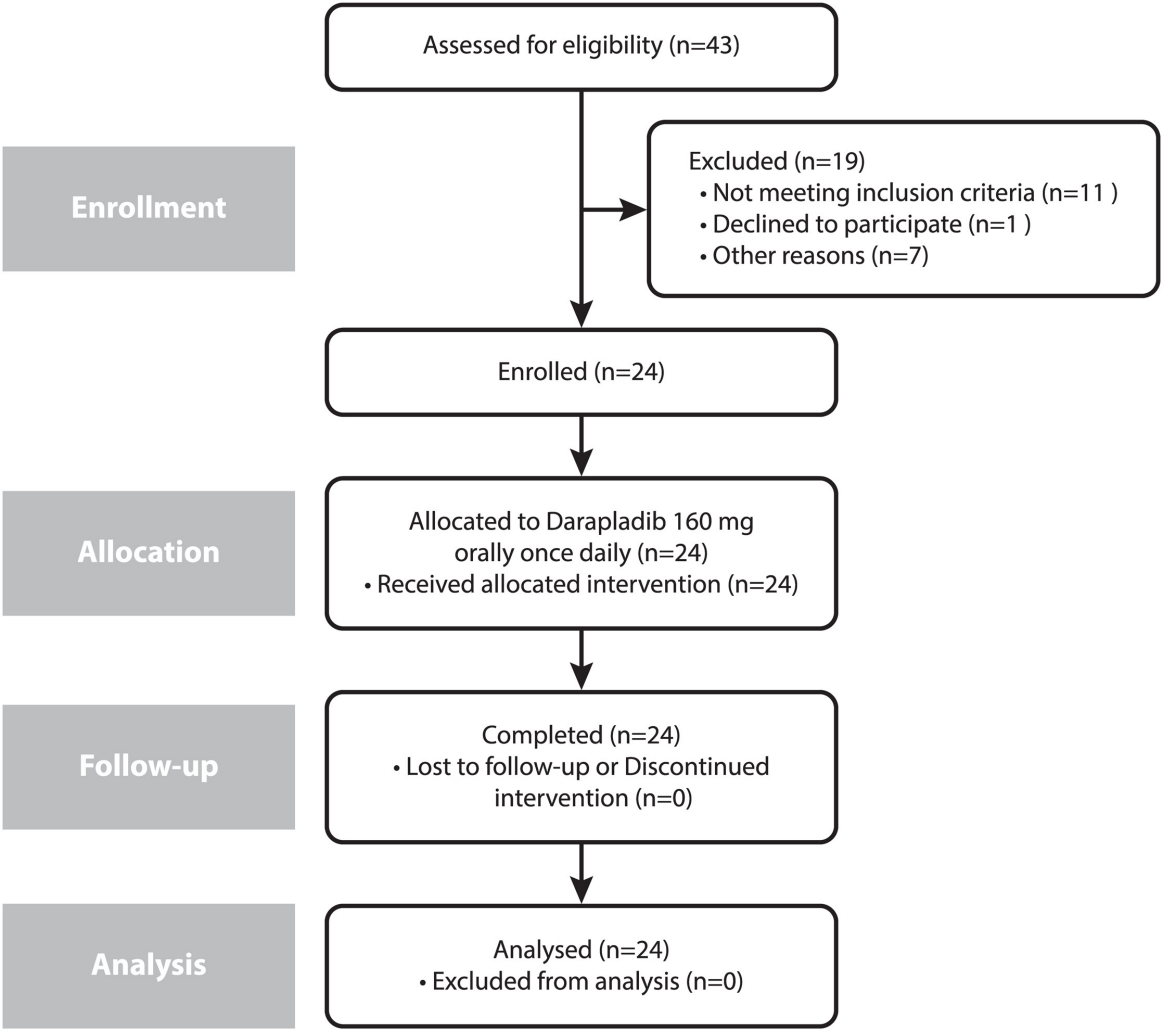
Subject disposition flow diagram where n is number of subjects in each category.

A total of 24 healthy Chinese subjects were enrolled in the study and received darapladib (Fig 1). All subjects completed the study. The study included subjects with mean age of 26 years (median 25.5, range 19–34), average weight of 63 kg (median 61, range 51–75), and body mass index of 22.5 kg/m^2^ (median 22.3, range 20.4–24.0) with an equal proportion of men and women (n = 12, each).

### Pharmacokinetics

**Fig 2:**
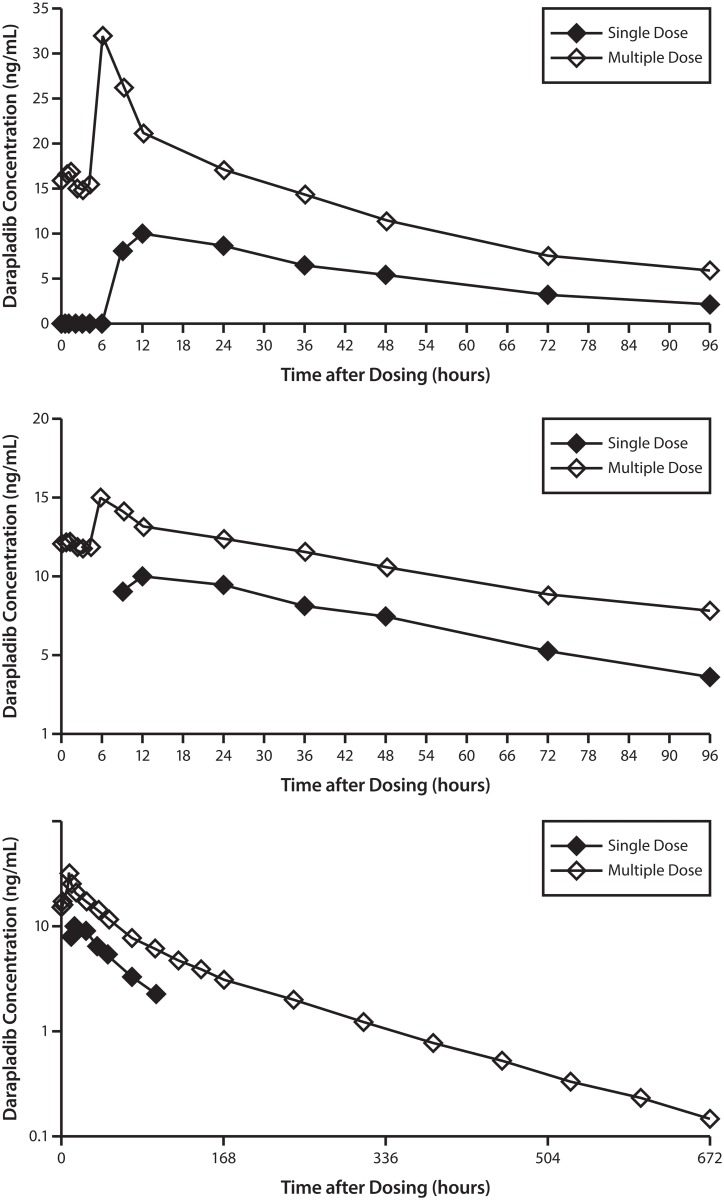
Median concentration-time profiles of darapladib following single and multiple dose administration over a period of 96 hours on linear-linear scale (A) and log- linear scale (B) truncated at 96 hours and (C) log-linear to the final observation.

The median plasma concentration-time profiles following single dose and multiple dose administration of darapladib are shown in Fig 2. The median time to attainment of Cmax was 9 hours following single dose administration and 6 hours following multiple dose administration (Table 1). The delay in the absorption of darapladib (absorption time lag of approximately 5–6 hour after single dose administration) (Fig 2A) is consistent with the enteric coated formulation. Overall, the absorption profile for darapladib following multiple dose administration was similar to that observed after single dose administration (Fig 2A). Following the peak, plasma concentrations declined slowly and the initial elimination rate to 96 hours was similar following single and multiple dosing (Fig 2B). The longer t½ of darapladib observed following multiple dose administration compared to single dose administration (Table 1) appears to be mainly associated with the terminal elimination phase that was characterized during the longer blood sampling following multiple than single dosing (Fig 2C).

**Table 1:** Pharmacokinetic parameters of darapladib following single and multiple dose administration of darapladib enteric coated tablet 160 mg once daily.

Parameters	Single Dose(N = 24)	Multiple Dose Day 28(N = 24)
C_max_ (ng/mL)^a^	17.9 (55.2)	34.4 (49.9)
T_max_ (hr)^b^	9.0 (6.0–24.0)	6.0 (0.0–15.0)
AUC_(0-τ)_ (ng.h/mL)^a^	153 (69.0)	519 (33.3)
AUC_(0-∞)_ (ng.h/mL)^a^	645 (34.0)	NA
t_1/2_ (hr)^a^	39.2 (18.0)	116 (15.8)
Cτ Day 14 (ng/mL)^a^	NA	16.6 (28.1)
Cτ Day 26 (ng/mL)^a^	NA	16.3 (26.2)
Cτ Day 27 (ng/mL)^a^	NA	16.6 (33.7)

^a^ Values are expressed as geometric mean (% geometric CV)

^b^ Values are expressed as median (range)

C_max_ = maximum plasma concentration; T_max_ = Time of occurrence of C_max_; AUC_(0-τ)_ = Area under the concentration-time curve over the dosing interval; AUC_(0-∞)_ = Area under the concentration-time curve from time zero (pre-dose) extrapolated to infinite time; t_1/2_ = Terminal phase half-life; Cτ = Pre-dose (trough) concentration; NA: Not applicable; N = Number of subjects.

As anticipated the systemic plasma exposure to darapladib (C_max_ and AUC_(0-T)_) following multiple dosing was higher than after single dose administration (Table 1). However, at Day 28 the observed accumulation ratio for AUC(0-T) (Ro = 3.38) was less than that predicted based on single dose data (R_p_ = 4.20) and the steady-state accumulation ratio was less than unity (Rs = 0.8), indicating that darapladib exhibits time-dependent pharmacokinetics (Table 2). Based on the assessment of pre-dose concentrations of darapladib, steady-state was reached by Day 14 of multiple dosing (Table 1).

**Table 2:** Accumulation ratios for darapladib and SB-553253 for multiple relative to single dosing of darapladib.

Parameter	Comparison	Ratio of GLS Means(90% CI)
**Darapladib**		
Ro	AUC_(0-τ)_ Day 28 vs AUC_(0-τ)_ Day 1	3.38 (2.67–4.28)
Rp	AUC_(0-∞)_ Day 1 vs AUC_(0-τ)_ Day 1	4.20 (3.54–5.00)
Rs	AUC_(0-τ)_ Day 28 vs AUC_(0-∞)_ Day 1	0.80 (0.71–0.92)
RC_max_	C_max_ Day 28 vs C_max_ Day 1	1.92 (1.62–2.27)
**SB-553253**		
Ro	AUC_(0-τ)_ Day 28 vs AUC_(0-τ)_ Day 1	1.47 (1.06–2.04)
RC_max_	C_max_ Day 28 vs C_max_ Day 1	1.46 (1.05–2.02)

Ro = Observed accumulation ratio; Rp = Predicted accumulation ratio; Rs = Steady-state accumulation ratio; RC_max_ = C_max_ accumulation ratio; AUC_(0-τ)_ = Area under the concentration-time curve over the dosing interval; AUC_(0-∞)_ = Area under the concentration-time curve from time zero (pre-dose) extrapolated to infinite time; CVw%: within-subject coefficient of variation; GLS mean = Geometric least square mean; NA: Not applicable

Systemic exposure to SB-553253 was lower than that of darapladib with median (SB-553253/ darapladib) ratios for AUC_(0-τ)_ of 0.0786 for single dose administration (Table 1) and 0.0532 for multiple dose administration (Table 3). The time to Cmax of SB-553253 was similar to that observed for darapladib. The t½ of SB-553253 following multiple dose administration was longer than that of darapladib; however, it was not possible to calculate t½ in the majority of subjects following single dose administration because the SB-553253 concentrations were not quantifiable for sufficient time interval.

**Table 3:** Pharmacokinetic parameters of metabolite SB-553253 following single and multiple once daily dosing of darapladib enteric coated tablet 160 mg. SB-553253 to Darapladib Cmax and AUC_(0-τ)_ ratios are also presented.

Parameters	N	Single Dose	N	Multiple Dose Day 28
C_max_ (ng/mL)^a^	23	1.97 (116.2)	24	2.79 (91.6)
T_max_ (hr)^b^	23	9.0 (6.0–24.0)	24	6.0 (0.0–15.0)
AUC_(0-t)_ (ng.h/mL)^a^	13	19.5 (99.7)	24	120 (57.3)
AUC_(0-τ)_ (ng.h/mL)^a^	10	19.4 (62.2)	24	26.5 (56.0)
t_1/2_ (hr)^a^	6^c^	19.4 (175.8)	19	251 (53.8)
Cτ Day 14 (ng/mL)^a^	-	NA	24	0.677 (43.3)
Cτ Day 26 (ng/mL)^a^	-	NA	24	0.656 (28.9)
Cτ Day 27 (ng/mL)^a^	-	NA	24	0.678 (33.7)
SB-553253 to darapladib AUC_(0-τ)_ ratio^b^	10	0.0786 (0.0445–0.2333)	24	0.0532 (0.0258–0.0939)
SB-553253 to darapladib C_max_ ratio^b^	23	0.1052 (0.0396–0.2391)	24	0.0742 (0.0270–0.1764)

^a^ Values are expressed as geometric mean (% between subject coefficient of variation)

^b^ Values are expressed as median (range)

^c^ In the majority of subjects the concentrations of SB553253 were generally not quantifiable for a sufficient duration of time to calculate t_½_

C_max_ = maximum plasma concentration; T_max_ = Time of occurrence of C_max_; AUC_(0-τ)_ = Area under the concentration-time curve over the dosing interval; AUC_(0-∞)_ = Area under the concentration-time curve from time zero (pre-dose) extrapolated to infinite time; t_1/2_ = Terminal phase half-life; Cτ = Pre-dose (trough) concentration; NA: Not applicable; N = Number of subjects

### Pharmacodynamics

Following multiple dosing of darapladib, steady-state trough (pre-dose) inhibition of plasma Lp-PLA_2_ enzyme activity was approximately 70% [70.3, 69.8, 69.6 and 71.4% on Days 14, 26, 27 and 28 respectively]. The mean maximum Lp-PLA_2_ enzyme inhibition at steady-state on Day 28 was 76.4% (Table 4). Lp-PLA_2_ enzyme activity returned to baseline approximately 19 days after the last dose of darapladib.

**Table 4:** Darapladib pharmacokinetic and pharmacodynamic parameters in healthy Chinese and Western subjects.

Parameter	Chinese	Western
Weight (kg)	63	78
AUC_(0-τ)_ (ng.h/mL) Day 1^a^	153 (69.0)	152 (118)
AUC_(0-∞)_ (ng.h/mL) Day 1^a^	645 (34.0)	560 (50.8)
AUC_(0-τ)_ (ng.h/mL) Day 28^a^	519 (33.3)	315 (40.5)
RCmax (90% CI)	1.92 (1.62–2.27)	0.94 (0.76–1.17)
Ro (90% CI)	3.38 (2.67–4.28)	2.08 (1.53–2.82)
Rp (90% CI)	4.20 (3.54–5.00)	3.70 (2.89–4.72)
Rs (90% CI)	0.80 (0.71–0.92)	0.56 (0.48–0.66)
Lp-PLA_2_ at baseline(nmol/min/mL) ^b^	89.3 (78.8–99.8)	172.0 (149–195)
Trough %Inhibition of Lp-PLA_2_ Day 28^b^	71.4 (68.7–74.0)	65.5 (63.3–67.7)
Peak %Inhibition of Lp-PLA_2_ Day 28^b^	76.4 (73.2–79.6)	72.5 (70.1–74.8)
IC_50_ (ng/mL)^a^	5.75 (7.72)	5.41 (5.27)

^a^ = Values are expressed as geometric mean [Coefficient of Variation %]

^b^ = Values are expressed as mean (95% Confidence Interval)

Ro = Observed accumulation ratio; Rp = Predicted accumulation ratio; Rs = Steady-state accumulation ratio; RC_max_ = C_max_ accumulation ratio; AUC_(0-τ)_ = Area under the concentration-time curve over the dosing interval; AUC_(0-∞)_ = Area under the concentration-time curve from time zero (pre-dose) extrapolated to infinite time; CI: Confidence Interval; IC50: darapladib plasma concentration causing 50% inhibition of plasma Lp-PLA2 activity. Data in Western subjects from separate study LPL112498 (Clinical Trial. Gov: NCT00743860).

### Pharmacokinetic/pharmacodynamic relationship

**Fig 3:**
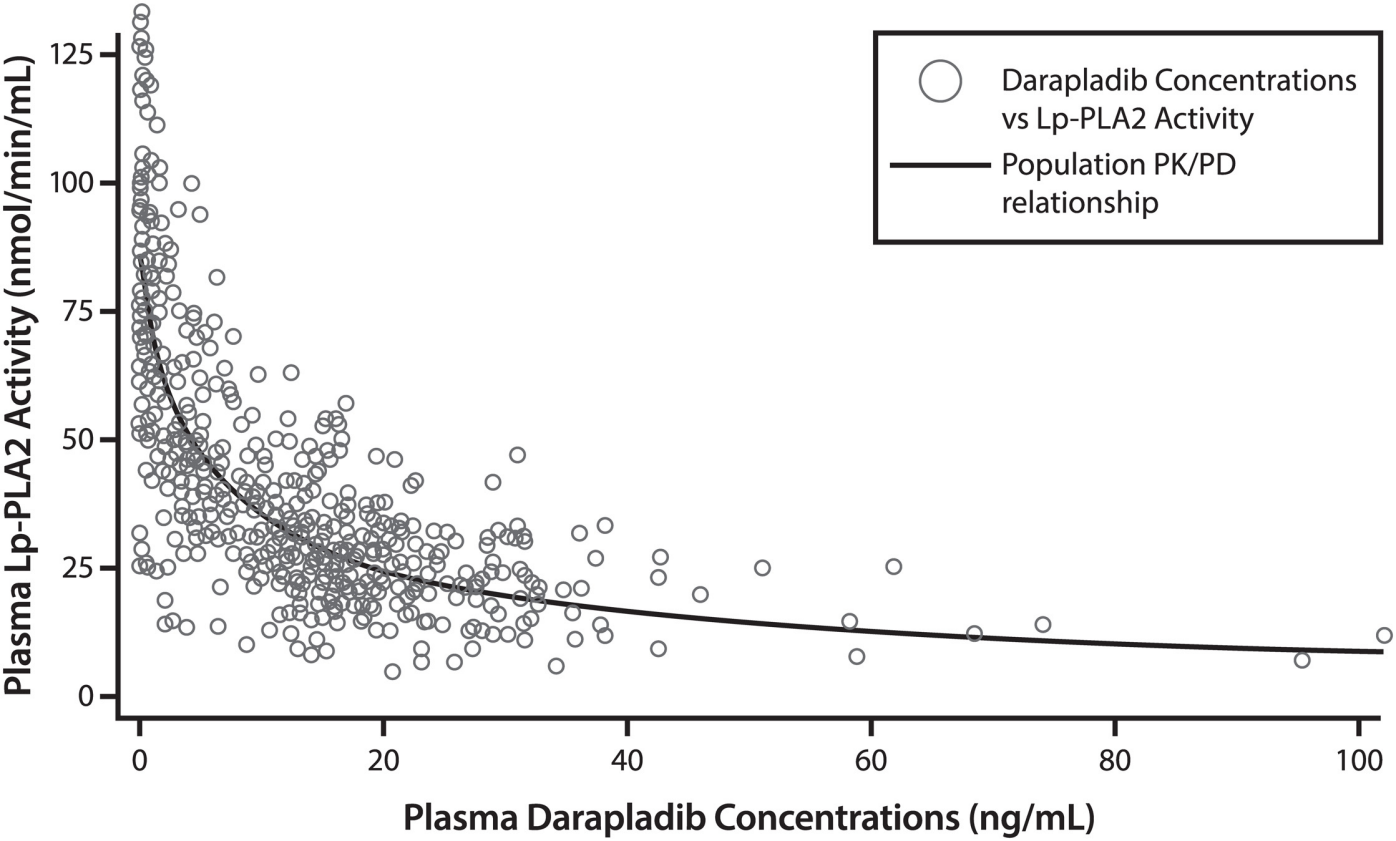
Plasma darapladib concentration versus Lp-PLA_2_ activity v after multiple dose of darapladib. Data points are indicated by symbols and the Emax relationship fitted is represented by the red line.

There was an inverse relationship between plasma Lp-PLA_2_ activity and plasma concentration of darapladib which was described by a sigmoid inhibitory E_max_ model (Fig 3). The PK-PD parameter estimates in the healthy Chinese subjects are described in Table 5.

**Table 5:** Emax model parameters for darapladib concentrations and associated LpPLA_2_ activity in healthy Chinese subjects.

Parameter	Pop. Mean (%CV*)	Inter-individual variability% ** (%CV*)
IC_50_ (ng/mL)	5.75 (7.72)	23.0 (40.7)
E_0_ (nmol/min/mL)	90.6 (5.25)	27.3 (33.2)
Hill coefficient (γ)	0.776 (3.48)	11.1 (38.7)
Residual variability** (%CV)	9.78 (13.8)	
Residual variability***	2.20 (22.4)	

* Precision expressed as coefficient of variation (CV).

** Expressed as % coefficient of variation.

*** Expressed as nmol/min/mL. IC_50_: darapladib plasma concentration causing 50% inhibition of plasma Lp-PLA_2_ activity. E_0_: baseline plasma Lp-PLA_2_ activity.

### Safety and tolerability

A total of 17/24 subjects (71%) experienced at least one TEAE during the study period. The most frequently (≥5 subjects [21%]) reported TEAEs were abnormal faeces, abnormal urine odour, diarrhea and nasopharyngitis. Fifteen subjects (63%) reported drug-related adverse effects and the most common drug-related AEs were abnormal faeces (8 subjects [33%]), abnormal urine odour (7 subjects [29%]), and diarrhoea (6 subjects [25%]). All the AEs were of mild intensity and resolved by the end of the study without any change in treatment. There were no clinically significant changes in laboratory values, vital signs, electrocardiograms (ECG) and physical examination. There were no serious AEs and no deaths were reported during the study.

## Discussion

This was the first study conducted in healthy Chinese subjects to characterise the pharmacokinetics, pharmacodynamic and safety of darapladib following single and multiple doses of the 160 mg enteric coated tablet.

The absorption of darapladib was slow, with an absorption lag time of approximately 5–6 hours (Fig 2A). The terminal phase half-life of darapladib estimated after multiple dose administration (116 hour) was longer than that estimated after single dose administration (39.2 hour), this is most likely a reflection of the longer duration of blood sample collection after multiple dosing (up to 28 days post- last dose) compared to single dose administration (4 days post-dose) rather than a true time dependent difference in the elimination of darapladib. This is supported by the observation that the elimination rate of darapladib is similar over the first 4 days after single and multiple dose administration (Fig 2B) Visual inspection of trough concentrations of darapladib indicated that steady-state had been reached by Day 14 of multiple dosing. However, it was not possible to determine when steady-state was achieved as pre-dose (trough) samples were not collected prior to Day 14.

**Fig 4:**
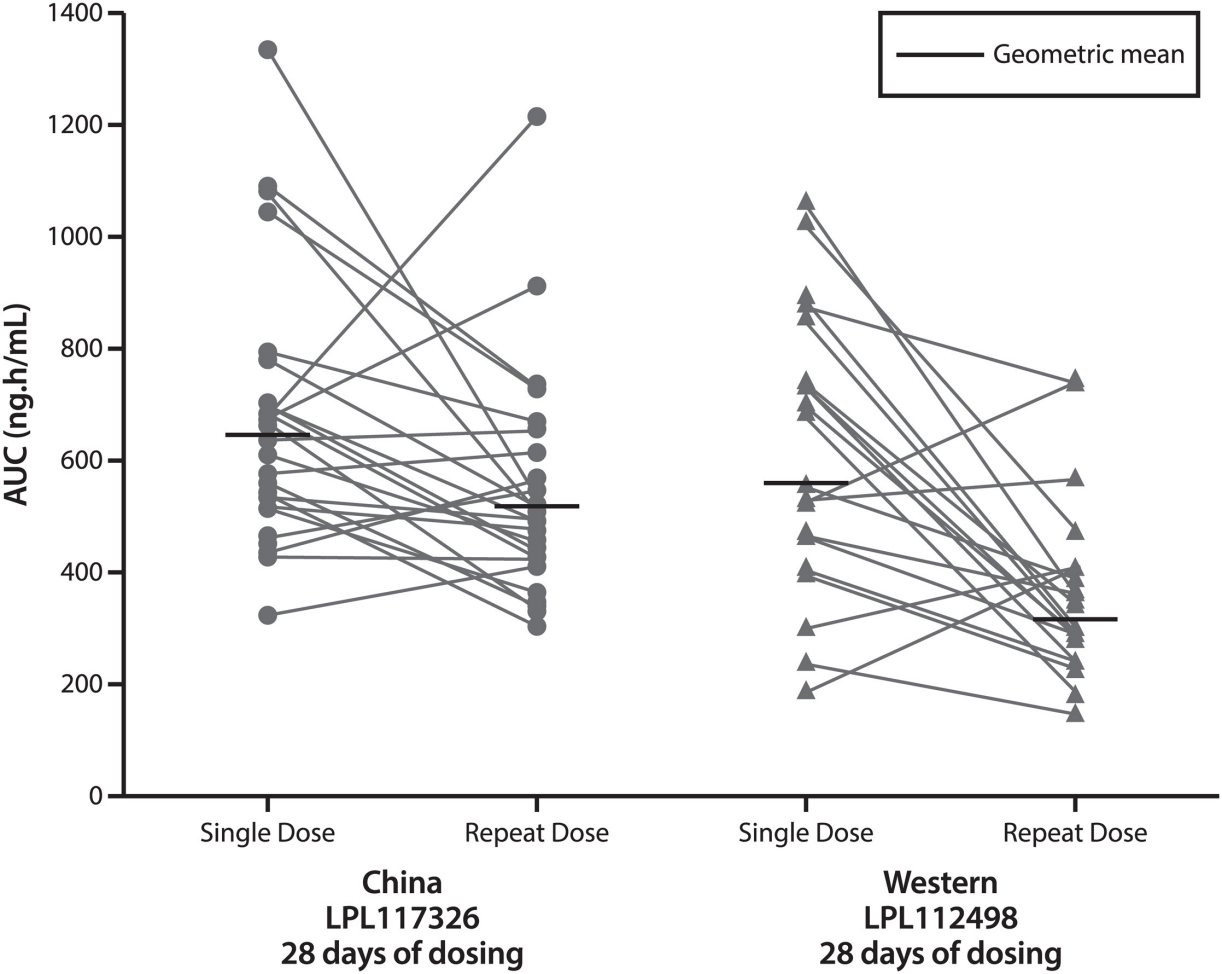
Darapladib single dose AUC_(0-∞)_ relative to repeat dose AUC_(0–24)_ in individual healthy Chinese and Western healthy subjects.

The systemic exposure (AUC_(0-∞)_) to darapladib following single dose administration in Chinese subjects was similar to previous observations in healthy Western subjects. However, the steady-state systemic exposure (AUC_(0-τ)_) was approximately 65% higher in the healthy Chinese subjects compared to healthy Western subjects (Fig 4). Since the higher systemic exposure in Chinese subjects only occurred following repeat dose and not following single dose administration, it is believed that the difference in pharmacokinetics between the two populations is due to differences in extrinsic factors during the repeat dose phase rather than an intrinsic inter-ethnic difference.

The Western data presented in Table 4 are from a healthy volunteer study of identical design to this study in healthy Chinese subjects. Both studies utilized the same darapladib formulation (enteric coated micronized drug product), the same darapladib dose (160 mg once daily), the same duration of multiple dosing (28 days) and all single and repeat doses were administered 1 hour after a “standard meal”. The only difference in study conduct identified was the composition of the “standard” breakfast consumed 1 hour prior to darapladib administration. The meals during the study, including breakfasts, were appropriate for each ethnic group. The breakfasts consumed were of a lower calorie content in the Chinese study (between 434 to 527 K) than in the Western study (between 571 to 1021 K) [Unpublished data].

The mechanism of the time dependent change in darapladib pharmacokinetics in Western subjects is not believed to be related to metabolic or elimination processes but is suspected to be related to a change (decrease) in the extent of darapladib absorption upon repeat dosing. A human radiolabel study [6] and a midazolam drug interaction study [7] indicate that the lower than expected accumulation of darapladib upon multiple dosing in Western subjects was not explained by induction of CYP3A4 mediated metabolism. In the healthy Chinese subjects the ratio of metabolite (SB-553253) to parent (darapladib) exposure at steady-state (AUC_(0-τ)_) (0.0532) is consistent with that observed in Western subjects (0.047) indicating no difference in the metabolic clearance of darapladib in Chinese and Western subjects upon repeat dosing. Since less than 0.5% of an oral dose of darapladib is recovered as unchanged darapladib in urine, the higher exposure to darapladib in Chinese subjects compared with Western subjects also cannot be explained by potential differences in renal clearance. The difference in the magnitude of the time dependent kinetics in Chinese relative to Western subjects is therefore most likely to be due to absorption differences upon multiple dosing. The low oral bioavailability of darapladib is driven by darapladib’s low passive permeability and low pH dependent solubility. It is plausible that differences in the diet could influence gastric pH levels and if the healthy Chinese subjects on repeat dosing were to have a slightly lower gastric pH, this may result in a slightly greater solubility and hence higher darapladib absorption and bioavailability relative to Western subjects. Even a small increase in bioavailability of darapladib in Chinese subjects could considerably increase overall systemic exposure darapladib compared to Western subjects. Such an increase in oral bioavailability is feasible given that in Western subjects the absolute bioavailability of darapladib in solution is 12% and of the enteric coated tablet is 4% [7]. However, it’s unclear why darapladib bioavailability would only be impacted in the healthy Chinese subjects following multiple dosing.

Despite the 65% higher systemic exposure to darapladib in healthy Chinese subjects following multiple doses, trough Lp-PLA_2_ activity (~70% inhibition) was marginally higher than observed in healthy Western (~66% inhibition) subjects. The Lp-PLA_2_ activity at baseline was lower in Chinese subjects (89.3 nmol/min/mL) than in Western subjects (172 nmol/min/mL). However, the IC_50_ determined from the Emax model in Chinese subjects (5.75 ng/mL) was similar to Western subjects (5.41 ng/mL) [Unpublished data]. Therefore an inter-ethnic difference in the sensitivity of LpPLA2 to darapladib was not observed. The higher darapladib concentrations at steady-state in Chinese than Western subjects are not associated with substantially greater effects on LpPLA2 activity and consequently would not be expected to require a dose adjustment in this ethnic group.

The safety data revealed no trends of clinical relevance in any of the safety parameters assessed in the healthy Chinese subjects. The adverse events observed in this study were consistent with those reported previously in Western subjects [Unpublished data].

The higher darapladib exposure in Chinese than Western subjects after multiple dose administration (mean AUC_(0-τ)_, 519 ng.h/mL) was still, on average, well below the No Observable Adverse Effect Level established in pre-clinical species (gender averaged AUC_(0-τ)_, 910.5 ng.h/mL) [Unpublished data]. The dose defining toxicity in the pre-clinical species was reversible phospholipidosis, an effect that is monitorable and in preclinical testing returns to baseline upon cessation of drug. Therefore, the exposure difference between Chinese and Western subjects observed in this study was not clinically significant.

## Conclusion

Although the systemic exposure to darapladib was higher in healthy Chinese than Western subjects on multiple dosing, the average exposure in Chinese subjects was well below the average No Observable Adverse Effect Level established in preclinical species. Additionally, the pharmacodynamic activity and safety profile in Chinese subjects were comparable to Western subjects. Therefore, ethnic-specific dose adjustment of darapladib is not considered necessary for the Chinese population.

## Supporting Information

S1 Protocol Study protocol. (PDF)

S1 TREND Checklist TREND Statement Checklist. (PDF)
