# Supplementary material for: Single and Multiple Dose Pharmacokinetics, Pharmacodynamics and Safety of the Novel Lipoprotein-Associated Phospholipase A2 Enzyme Inhibitor Darapladib in Healthy Chinese Subjects: An Open Label Phase-1 Clinical Trial
Source: PLoS One. 2015 Oct 14;10(10):e0139862. doi: 10.1371/journal.pone.0139862 (PMC4605839; doi:10.1371/journal.pone.0139862)
Supplement: S1 Protocol — (PDF) [file pone.0139862.s001.pdf]

TITLE PAGE

Division: Worldwide Development
Retention Category: GRS019
Information Type: Protocol Amendment

**Title:**	A Study to Evaluate the Pharmacokinetics, Pharmacodynamics and Safety of 160 mg Enteric-coated Micronised Free Base Darapladib in Healthy Chinese Subjects.

Compound Number: SB-480848

Effective Date: 30-SEP-2013

Protocol Amendment Number: 02

Subject: Darapladib, Safety, Pharmacokinetics, Pharmacodynamics, Healthy Chinese Subjects

Author: Julia Zhu, Yi Liu, Jack Peng, Jiansong Yang

Copyright 2013 the GlaxoSmithKline group of companies. All rights reserved. Unauthorised copying or use of this information is prohibited.

Revision Chronology:

**2012N152694_00**	2013-May-20	Original
**2012N152694_01**	2013-Jul-31	Amendment No.: 01<br><br>Change the medical monitor from Julia Zhu to Ray Tao<br><br>Modify the target subject number for analysis from 20 to 18<br><br>Modify the pregnancy test procedure of inclusion criteria<br><br>Modify the exclusion criteria to exclude subject with syphilis antibody positive<br><br>Clarify pregnancy test procedure and Sampling, ie, blood test at Screening Visit<br><br>Add Chest X ray examination at Day -1 to exclude subject with clinical significant abnormalities<br><br>Clarify the SAE assessments and report procedure for Screen and Baseline failure<br><br>Delete Instructions for medical device use<br><br>Update time and events table according to related inclusion and exclusion criteria changing and typo correction. Specify the Hema/Chem/Urinalysis tests exemption at Day -1 to avoid duplication.<br><br>Update Clinical Laboratory Assessments according to changes in exclusion criteria<br><br>Delete Cardiovascular Event and Death Events collection according to new process of Cardiovascular Event & Death eCRF initiative<br><br>Recalculate the Sample Size Assumptions<br><br>Typo correction in Appendix 2<br><br>Clarify no health outcomes questionnaires

**2012N152694_02**	2013-Sep-30	Amendment No.: 02 Update and correction of different items in protocol.<br><br>Updated the safety monitoring scheme alter approval process<br><br>Revise the blood sampling volume. “4 mL” of blood for haematological and chemical test. “3 mL” of blood for virological assessment. “2 ml” blood for blood pregnancy test at screening for female.<br><br>Add more information about potential additional blood loss “10 ml” in blood sampling procedure<br><br>Typo correction in Time Event Table From “Blood β-HCG test” to “Blood hCG test”

SPONSOR SIGNATORY

Sept 30, 2013

Min Irwin MD

Date

VP, Medicines Development

GlaxoSmithKline (China) R&D

SPONSOR/MEDICAL MONITOR INFORMATION PAGE

Medical Monitor and Sponsor Contact Information:

**Role**	Name	Day Time Phone Number	After-hours Phone/Cell/ Pager Number	Fax Number	GSK Address
Primary Medical Monitor	Ray Tao	+86 (0)21 6159 0868	+86 139 1788 7601	+86 (0)21 6159 0705	GSK R&D China<br>No.1 building, 917 Halei Road<br>Zhangjiang Hi-tech Park,<br>Pudong
Secondary Medical Monitor	Yi Liu	+86 (0)21 61590560	+86 186 1617 6990	+86 (0)21 6159 0705	GSK R&D China<br>No.1 building, 917 Halei Road<br>Zhangjiang Hi-tech Park,<br>Pudong
SAE fax number				+86 (0) 21 5895 2078	

Sponsor Legal Registered Address:

GlaxoSmithKline Research & Development Limited
No. 1 building, 917 Halei Road
Zhangjiang Hi-tech Park, Pudong
Shanghai, 201203, China

In some countries, the clinical trial sponsor may be the local GlaxoSmithKline affiliate company (or designee). If applicable, the details of the alternative Sponsor and contact person in the territory will be provided to the relevant regulatory authority as part of the clinical trial application.

Regulatory Agency Identifying Number(s): clinical trial permit number 2009L05633

INVESTIGATOR PROTOCOL AGREEMENT PAGE

I confirm agreement to conduct the study in compliance with the protocol, as amended by this protocol amendment.

Investigator Name:		
Investigator Address:		
Investigator Phone Number:		
Investigator Signature		Date

TABLE OF CONTENTS

PAGE

LIST OF ABBREVIATIONS 10

1. INTRODUCTION 13
   1.1. Study Rationale 13
   1.2. Brief Background 13

2. OBJECTIVE(S) AND ENDPOINT(S) 16

3. STUDY DESIGN 17
   3.1. Study Design Detail 17
   3.2. Discussion of Study Design 17
      3.2.1. Design Rationale 17
      3.2.2. Dose Rationale 17
   3.3. Risk Management 18
      3.3.1. 4-Fluorobenzyl chloride (4-FBCl) 18
      3.3.2. Theoretical Concern Regarding PAF Accumulation with Lp-PLA_2 Inhibitors 19
      3.3.3. Ongoing Rat and Mouse, 2-Year (“Lifetime”) Carcinogenicity Studies 19

4. STUDY POPULATION 22
   4.1. Number of Subjects 22
   4.2. Eligibility Criteria 22
      4.2.1. Inclusion Criteria 22
      4.2.2. Exclusion Criteria 23
         4.2.2.1. Criteria Based Upon Medical Histories 23
         4.2.2.2. Criteria Based Upon Diagnostic Assessments 24
         4.2.2.3. Other Criteria 24
   4.3. Lifestyle and/or Dietary Restrictions 24
      4.3.1. Contraception Requirements 24
         4.3.1.1. Female Subjects 24
      4.3.2. Meals and Dietary Restrictions 25
      4.3.3. Caffeine, Alcohol, and Tobacco 25
      4.3.4. Activity 26
   4.4. Screen and Baseline Failures 26
   4.5. Withdrawal Criteria and Procedures 26
   4.6. Subject Completion 26

5. STUDY TREATMENT 27
   5.1. Investigational Product 27
   5.2. Treatment Assignment 27
   5.3. Subject Specific Dose Adjustment/Stopping Criteria 27
      5.3.1. Liver Chemistry Stopping Criteria 27
      5.3.2. QTc Withdrawal Criteria 27
      5.3.3. Other Dose Adjustment/Stopping Safety Criteria 28
   5.4. Blinding 28
   5.5. Packaging and Labeling 28
   5.6. Preparation/Handling/Storage/Accountability 28
   5.7. Assessment of Compliance 29

5.8. Treatment of Study Treatment Overdose 29
5.9. Treatment After the End of the Study 29
5.10. Concomitant Medications and Non-Drug Therapies 29
5.10.1. Permitted Medications 29
5.10.2. Prohibited Medications and Non-Drug Therapies 30

6. STUDY ASSESSMENTS AND PROCEDURES 31
6.1. Time and Events Table 32
6.2. Demographic/Medical History Assessments 36
6.3. Safety 36
6.3.1. Physical Exams 36
6.3.2. Vital Signs 36
6.3.3. Electrocardiogram 36
6.3.4. Clinical Laboratory Assessments 36
6.4. Pharmacokinetics 37
6.4.1. Blood Sample Collection 37
6.4.2. Sample Analysis 38
6.5. Biomarkers/Pharmacodynamic Markers 38
6.5.1. Confirmed Biomarkers/Pharmacodynamic Markers 38

7. ADVERSE EVENTS , SERIOUS ADVERSE EVENTS , PREGNANCY 39
7.1. Adverse Events and Serious Adverse Events 39
7.1.1. Time period for collecting AE and SAE information 39
7.1.2. Definition of Adverse Events 39
7.1.3. Definition of Serious Adverse Events 40
7.1.4. Prompt Reporting of SAEs to GSK 42
7.1.5. Regulatory Reporting Requirements for SAEs 42
7.2. Pregnancy 42
7.2.1. Time period for collecting pregnancy information 42
7.2.2. Action to be taken if pregnancy occurs 42
7.2.3. Action to be taken if pregnancy occurs in a female partner of a male study Subject 43

8. DATA ANALYSIS AND STATISTICAL CONSIDERATIONS 44
8.1. Hypotheses and Treatment Comparisons 44
8.2. Sample Size Considerations 44
8.2.1. Sample Size Assumptions 44
8.2.2. Sample Size Sensitivity 45
8.2.3. Sample Size Re-estimation 45
8.3. Data Analysis Considerations 45
8.3.1. Interim Analysis 45
8.3.2. Final Analyses 45
8.3.2.1. Analysis Populations 45
8.3.2.2. Safety Analyses 45
8.3.2.3. Pharmacokinetic Analyses 46
8.3.2.4. Pharmacodynamic/Biomarker Analyses 47
8.3.2.5. Pharmacokinetic/Pharmacodynamic Analyses 47

9. STUDY GOVERNANCE CONSIDERATIONS 48
9.1. Posting of Information on Publicly Available Clinical Trial Registers 48
9.2. Regulatory and Ethical Considerations, Including the Informed Consent Process 48

9.3. Quality Control (Study Monitoring) ... 48
9.4. Quality Assurance ... 49
9.5. Study and Site Closure ... 49
9.6. Records Retention ... 49
9.7. Provision of Study Results to Investigators, Posting of Information on Publically Available Clinical Trials Registers and Publication ... 50

10. REFERENCES ... 51

11. APPENDICES ... 52
11.1. Appendix 1: Liver Safety Process ... 52
11.2. Appendix 2: Procedures for Detection, Evaluation, Follow-Up and Reporting of Adverse Events ... 55
11.3. Appendix 3: Clinical Criteria for Diagnosing Anaphylaxis ... 58
11.4 Appendix 4: Protocol Amendment Changes ... 59

LIST OF ABBREVIATIONS

ACS	Acute coronary syndrome
AE	Adverse Event
ALT	Alanine aminotransferase (SGPT)
ALP	Alkaline phosphatise
ANOVA	Analysis of Variance
AST	Aspartate aminotransferase (SGOT)
AUC	Area under concentration-time curve
AUC(0-∞)	Area under the concentration-time curve from time zero (pre-dose) extrapolated to infinite time
AUC(0-t)	Area under the concentration-time curve from time zero (pre-dose) to last time of quantifiable concentration
AUC(0-τ)	Area under the concentration-time curve over the dosing interval
BP	Blood pressure
BUN	Blood urea nitrogen
CHD	Coronary heart disease
CI	Confidence Interval
Cmax	Maximum observed concentration
Cmin	Minimum observed concentration
Cτ	Pre-dose (trough) concentration at the end of the dosing interval
Ct	Last observed quantifiable concentration
CO_2	Carbon dioxide
CPMS	Clinical Pharmacokinetics Modelling & Simulation
CPSR	Clinical Pharmacology Study Report
CP-RAP	Clinical Pharmacology Reporting and Analysis Plan
CRF	Case Report Form
CV	Coefficient of variance
DBP	Diastolic blood pressure
DME	Diabetic macular edema
DMPK	Drug Metabolism and Pharmacokinetics
EC	Enteric-coated
ECG	Electrocardiogram
EDTA	Ethylenediamine tetraacetic acid
E0	Zero effect dose
FDA	Food and Drug Administration
FEV1	Forced expiratory volume in 1 second
4-FBCl	4-fluorobenzyl chloride
FSH	Follicle stimulating hormone
GCP	Good Clinical Practice
GGT	Gamma glutamyltransferase
GLP	Good Laboratory Practice
GSK	GlaxoSmithKline
HBsAg	Hepatitis B surface antigen
hCG	Human chorionic gonadotropin
HIV	Human Immunodeficiency Virus

h/hr	Hour(s)
HR	Heart rate
IB	Investigator’s Brochure
IC50	Half maximal inhibitory concentration
ICH	International Conference on Harmonization of Technical Requirements for Registration of Pharmaceuticals for Human Use
IDMC	Independent Data Monitoring Committee
IDSL	Integrated Data Standards Library
IEC	Independent Ethics Committee
IgM	Immunoglobulin M
INR	International Normalized Ratio
IP	Investigational Product
IRB	Institutional Review Board
IU	International Unit
Kg	Kilogram
L	Liter
LDL	Low density lipoprotein
LFTs	Liver function tests
Lp-PLA$_2$	Lipoprotein-associated phospholipase A$_2$
LSLV	Last Subject’s last visit
Lyso-PC	Lysophosphatidylcholine
µg	Microgram
µL	Microliter
MCH	Mean corpuscular hemoglobin
MCHC	Mean corpuscular hemoglobin concentration
MCV	Mean corpuscular volume
Mg	Milligrams
mL	Milliliter
MSDS	Material Safety Data Sheet
msec	Milliseconds
PAF	Platelet-activating factor
PAF-AH	Platelet-activating factor acetyl hydrolase
PC20	Provocative concentration of methacholine causing a 20% fall in FEV1
PD	Pharmacodynamic
PIMS	Phase I Management System
PK	Pharmacokinetic
QC	Quality control
QD	Once daily
QTc	Corrected Q-T interval
QTcB	QT duration corrected for heart rate by Bazett’s formula
QTcF	QT duration corrected for heart rate by Fridericia’s formula
γ	Gradient coefficient
RAP	Reporting and Analysis Plan
RBC	Red blood cells
Rcmax	Cmax accumulation ratio
Ro	Observed accumulation ratio

Rp	Predicted accumulation ratio
Rs	Steady-state accumulation ratio
SAE	Serious adverse event(s)
SAS	Statistical Analysis Software
SD	Standard deviation
SGOT	Serum glutamic-oxaloacetic transaminase
SGPT	Serum glutamic pyruvic transaminase
SOP	Standard Operating Procedure
SPM	Study Procedures Manual
T	Infusion duration
t	Time of last observed quantifiable concentration
t½	Terminal phase half-life
τ	Dosing interval
tmax	Time of occurrence of Cmax
ULN	Upper limit of normal
WBC	White blood cells

Trademark Information

Trademarks of the GlaxoSmithKline group of companies
NONE

Trademarks not owned by the GlaxoSmithKline group of companies
WinNonlin

1. INTRODUCTION

1.1. Study Rationale

Darapladib is a novel selective and orally active inhibitor of lipoprotein-associated phospholipase A_2 (Lp-PLA_2) in development for the treatment of atherosclerosis.

Globally, 27 clinical pharmacology studies were conducted with single and repeat oral doses of up to 100 mg of the non-enteric coated free base formulation and up to 480 mg of the enteric-coated (EC) free base micronised formulation have been administered. Two other Phase II studies in Subject with stable coronary heart disease (CHD), CHD risk or acute coronary syndrome (ACS) have been recently completed and shown significant and sustained inhibition of plasma Lp-PLA_2 activity and good safety and tolerability as well. There are 2 ongoing randomized, parallel group, placebo-controlled, event-driven Phase III studies in patients with CHD and ACS. In addition, darapladib is also being developed for an ongoing Phase II clinical trial in the diabetic macular edema (DME) indication.

The study is designed to assess the pharmacokinetics (PK) of darapladib and its metabolite SB-553253 in healthy Chinese Subjects. SB-553253 is the major metabolite with pharmacological activity, which has been quantified in all preclinical toxicity studies, as well as in all clinical studies. Plasma Lp-PLA_2 activity will also be analyzed to assess the time course of enzyme activity following repeat dosing and during the off-drug period.

Darapladib is being developed for the treatment of atherosclerosis in China; therefore, this study is being conducted to provide safety, tolerability, PK and pharmacodynamic (PD) data that will support studies with darapladib in Chinese patients.

1.2. Brief Background

Darapladib has potential for the treatment of atherosclerosis. The compound was selected on the basis of its potency and efficacy, both in vitro and in vivo, as an inhibitor of Lp-PLA_2 in animal models.

Cardiovascular (CV) disease accounts for approximately 50% of all deaths from myocardial infarction and stroke in both developed and developing countries. Atherosclerosis is the fundamental pathology behind most CV deaths and is now universally acknowledged as an inflammatory disease and not one that simply results from the arterial accumulation of lipids. This specialized inflammatory disease of the intima is characterized by leukocyte (monocytes and T-lymphocytes) recruitment and accumulation having a major role in both early and continuing aspects of its pathogenesis.

Thus, macrophage driven chronic inflammation appears to represent an important destabilizing process in the arterial lesion [Ross, 1999]. The sub-endothelial oxidation of low density lipoprotein (LDL) is viewed as a highly significant biological process that both initiates and accelerates arterial lesion development [Ross, 1999]. One of the earliest events in LDL oxidation is the hydrolysis of oxidatively modified phosphatidylcholine, generating substantial quantities of lysophosphatidylcholine (lyso-PC) and oxidized fatty acids. This hydrolysis is mediated by Lp-PLA_2, an enzyme that is associated predominantly with LDL in human plasma [Tew, 1996].

Lp-PLA_2 is also referred to in the literature as platelet-activating factor acetyl hydrolase (PAF-AH), since it was first characterized as a plasma enzyme activity that hydrolysed exogenously added platelet-activating factor (PAF). Several lines of evidence indicate that both by-products of Lp-PLA_2-mediated hydrolysis of oxidized LDL (i.e. lyso-PC and oxidized fatty acids) are pro-inflammatory and atherogenic mediators [Macphee, 1999]. Thus, preventing the generation of these mediators through inhibition of Lp-PLA_2 should retard atherosclerosis by interfering with inflammatory cell localization, activation and pro-inflammatory function. Consistent with this notion is the recent observation that plasma levels of Lp-PLA_2 represent an independent predictor of CHD [Packard, 2000; Garza, 2007].

Darapladib may prevent the accumulation of lyso-PC and other pro-inflammatory lipids generated during the oxidation of LDL by inhibiting the Lp-PLA_2 enzyme. As the approach specifically targets the underlying chronic inflammation, an inhibitor of Lp-PLA_2, such as darapladib, is predicted to inhibit intimal macropharge accumulation and activation and therefore, to retard and stabilize the developing atherosclerotic plaque. As such, darapladib has important potential for chronic treatment of atherosclerosis.

Clinical experience with darapladib to date

To date, 27 Phase I studies have completed, with 652 healthy Subjects, 34 Subjects with asthma, and 12 Subjects with moderate hepatic impairment exposed to at least 1 dose of darapladib. In 5 completed Phase II studies, 1133 patients have received darapladib for periods of up to 12 months, and 383 Subjects have received darapladib EC tablets 160 mg once daily for 12 weeks or longer.

Currently, 2 double-blind Phase III studies are ongoing. Study LPL100601 and Study 480848/033 are 2 double-blind, randomized, placebo-controlled Phase III outcomes studies which designed to evaluate the clinical efficacy of long-term treatment with darapladib EC tablets, 160 mg (oral once daily [QD] dose) when added to standard of care in a chronic CHD population and an ACS patient population, respectively. There were 15,828 and 13,027 Subjects randomized in Study LPL100601 and in Study 480848/033. China has participated in the clinical development of the 2 international multi-center trials, and contributed 813 Subjects to these trials.

Lipoprotein-associated phospholipase A_2 (Lp-PLA_2) has also been postulated to play an important role in diabetes-induced vascular leakage. Inhibition of Lp-PLA_2 activity through oral administration of darapladib may lead to reduction of inner blood-retinal barrier breakdown and thus provide another treatment option. A phase II study in the DME indication is designed to evaluate whether inhibition of Lp-PLA_2 activity through oral administration of darapladib may lead to improvements in retinal edema and visual acuity. Study recruitment is ongoing, with a total of 27 Subjects randomized before June 2012.

To date, darapladib has been well tolerated. The most common adverse events (AEs) following both single and repeat dose administration of darapladib have been headache, abnormal urine (abnormal odour), diarrhoea and abdominal pain. The potential for darapladib to be associated with reports of odour and taste-related AEs was identified among volunteers, with the report frequency varying widely among studies (range of approximately 15-63%). These odour-related AEs include taste perversion as well as an

abnormal odour of feces, urine, sweat, body and/or hair. There have been no withdrawals due to odour-related AEs.

2. OBJECTIVE(S) AND ENDPOINT(S)

Objectives	Endpoints
**Primary**	
1. To assess the safety and tolerability of single and repeat oral dose of 160 mg EC darapladib in healthy Chinese Subjects.<br>2. To assess the primary PK parameters of single and repeat oral dose of 160 mg EC darapladib in healthy Chinese Subjects.	1. Clinical safety data from spontaneous AE reporting, 12-lead electrocardiogram (ECG) recording, vital sign measurement, nursing/physician observation and clinical laboratory examination will be summarized and any clinically significant abnormalities described.<br>2. The primary PK endpoints of interest are area under the concentration-time curve from time zero (pre-dose) to last time of quantifiable concentration (AUC[0-t]), area under the concentration-time curve from time zero (pre-dose) extrapolated to infinite time (AUC [0-∞]), maximum observed concentration (C_{max}) and accumulation ratios (C_{max} accumulation ratio [Rc_{max}] and observed accumulation ratio [Ro]) of darapladib.
**Secondary**	
1. To assess the secondary PK parameters of single and repeat oral dose of 160 mg EC darapladib and the PK parameters of the pharmacologically active metabolite SB-553253 in healthy Chinese Subjects.<br>2. To evaluate the inhibition of plasma Lp-PLA2 activity after repeat oral doses of 160 mg EC darapladib in healthy Chinese Subjects.	1. The secondary PK endpoints of interest are time of occurrence of C_{max} (T_{max}) and terminal phase half-life (T1/2) of darapladib and AUC(0-t), AUC(0-∞), Cmax, T1/2 and Tmax of the pharmacologically active metabolite SB-553253 (as data permit).<br>2. Plasma Lp-PLA2 activity, expressed in terms of percent inhibition relative to baseline.
**Exploratory**	
To describe the plasma concentration-Lp-PLA_2 activity inhibition relationship following repeat oral doses of 160 mg EC darapladib.	Estimation of PK/PD parameters [such as half maximum inhibitory concentration (IC50), Zero effect dose (E0), gradient coefficient (γ)] and their associated variability, if data permit.

3. STUDY DESIGN

3.1. Study Design Detail

This will be an open label study where each Subject will participate in 2 study sessions (single and repeat dose). All Subjects will receive 160 mg EC micronised free-base darapladib as a single dose and as repeated daily dose for 28 days. There will be at least 4 days between dosing in the Single Dose Session and the first dose of the Repeat Dose Session due to the 96-hour PK sampling period following the single dose. PK samples will be collected over a 96-hour period after single dose in Session 1, and over a 24-hour period after Day 28 of repeat dosing, as well as over a 28-day duration following the last day of dosing of the repeat dose session. PD samples will be collected over a 24-hour period after Day 28 of repeat dosing, as well as over the 28-day duration following the last day of dosing of the repeat dose session.

Subjects will return approximately 28 days after the last dose of study medication for a follow-up visit. The total study duration for each Subject including the Screening, Treatment and Follow-up periods will be approximately 12 weeks.

3.2. Discussion of Study Design

3.2.1. Design Rationale

The study is designed to investigate the PK profiles of darapladib 160 mg after single dose and repeat dose in healthy Chinese Subjects. The study is open-label as the primary endpoint will not be influenced by Subjects knowing which medication they are receiving. The Subjects will have PK samples collected for more than 5 half-lives after dosing to enable a comprehensive PK profile to be generated. The design employed for this study is identical to the design of a previous Phase I study conducted on healthy western Subjects (Protocol Number LPL112498).

3.2.2. Dose Rationale

As of December 2011, a total of approximately 652 healthy volunteers and 1179 patients from completed clinical trials had received darapladib. In overseas studies single and repeat oral QD doses of up to 100 mg of the non-EC freebase formulation and up to 480 mg of the EC free base micronised formulation of darapldib have been administered. Darapladib has been well tolerated. Mean half-life of darapladib was 126 hours following QD dosing of 160 mg EC tablets of micronised free-base darapladib to healthy western Subjects (Protocol Number LPL112498). Darapladib is expected to reach its steady state following repeat dosing of 28 days.

Study LPL110736, a double blind, randomized, parallel, placebo-controlled study, was conducted in Japan to investigate the safety, tolerability, PK and PD following 160 mg enteric coated, micronized free-base daily doses of darapladib in healthy Japanese male Subjects for 12 days. A substantial inter-ethnic difference in darapladib PK and PD was not observed.

Based on these data, this study will be conducted to determine the safety/tolerability, PK and PD of the EC, free base micronised darapladib 160 mg in the single dose session and repeat dose session in healthy Chinese Subjects. EC free base (micronised) darapladib 160 mg in this study is currently being evaluated in the ongoing global phase III studies of darapladib.

Darapladib will be administered as EC tablets formulated to begin disintegration at pH 5.5 and above. Under these conditions, the formation of 4-fluorobenzyl chloride, a potential alkylating agent that is an acid degradation product of darapladib that is formed only at very low pH in the presence of chloride ions, is anticipated to be negligible at this proposed dose.

3.3. Risk Management

3.3.1. 4-Fluorobenzyl chloride (4-FBCl)

4-fluorobenzyl chloride (4-FBCl), a potential acid degradant of darapladib, is a weak genotoxin in-vitro [Darapladib Investigator's Brochure, Version 09, GlaxoSmithKline Document Number RM2003/00513/06]. The data from genotoxicity assessments suggest that darapladib itself does not present a genotoxic hazard to humans and that, when darapladib enteric coated tablets, are taken in accordance with instructions, the genotoxic risk associated with potential trace levels of the acid degradant 4-FBCl is minimal. The early in vitro experiments showed that , based on an extrapolation from experimentally observed values, a maximum of approximately 160 ug 4-FBCl could be formed per 1g darapladib in simulated gastric fluid (pH 1.2) after 4 hours at 35 to 40 °C. This could result in a theoretical maximum human dose of 26 ug 4-FBCl from a 160 mg tablet (0.52 ug/kg to a 50 kg individual). Importantly, further experimentation in simulated gastric fluid at pH 2, 3 and 4 has been performed and 4-FBCl was not detected (limit of detection 1ug/g). This demonstrates that 4-FBCl is only detected at pH less than 2; pH values of less than 2 are typically only encountered in the fasted state. Based on preclinical data, the level of containment provided by enteric coating, and the low maximum potential exposure in the rare case where the enteric coat might fail, it is concluded that there is minimal genotoxic risk associated with the potential trace levels of 4-FBCl that might result from oral administration of darapladib.

In addition, subjects will be instructed that darapladib tablets must be swallowed whole and not chewed, to maintain the integrity of the enteric coat. Further, subjects will be instructed to take the tablets after eating a meal in order to raise the pH of the stomach and provide additional protection in the rare event that drug is released in the stomach.

For additional information, refer to the IB [Darapladib Investigator's Brochure, Version 09, GlaxoSmithKline Document Number RM2003/00513/06]. Investigators will be instructed to ensure that subjects understand and comply with dosing instructions.

3.3.2. Theoretical Concern Regarding PAF Accumulation with Lp-PLA_2 Inhibitors

Theoretical concern of bronchospasm with Lp-PLA_2 inhibition

All of the side effects of darapladib may not be known. Based upon mechanism of action, inhibition of Lp-PLA_2 may contribute to PAF accumulation, which could contribute to bronchospasm.

A specific study to evaluate the effect of darapladib in patients with asthma (LPL107629) showed no statistically or clinically significant effect of darapladib on FEV1 (forced expiratory volume in 1 second) following single day (Day 1) and repeat oral doses (Day 21), and no statistically significant treatment effect observed on PC20 (provocative concentration of methacholine causing a 20% fall in FEV1) at trough Day 21. However, as a precautionary measure, subjects with history of severe asthma that is poorly-controlled by pharmacotherapy are excluded from clinical trials with darapladib. For additional information refer to the IB [Darapladib Investigator’s Brochure, Version 09, GlaxoSmithKline Document Number RM2003/00513/06].

Theoretical concern of increasing severity of anaphylaxis with Lp-PLA_2 inhibition

A study suggested that PAF is positively correlated and PAF-AH is inversely correlated with anaphylaxis severity [Vadas, 2008]. A separate retrospective analysis showed that PAF-AH levels were significantly lower in patients with fatal peanut anaphylaxis than those with mild allergic reactions to peanuts and subjects in the control group. However, the exact association between PAF-AH level and the risk of increasing severity of anaphylaxis is unknown.

As a precautionary measure, subjects with history of anaphylaxis (refer to Appendix 3) or severe allergy (e.g., due to food, medications, or latex) as defined by the summary report of the second symposium on the definition and management of anaphylaxis [Sampson, 2006] are excluded from clinical studies with darapladib.

Events of anaphylaxis and severe allergy in this patient population will continue to be monitored by the sponsor. For additional information refer to the IB [Darapladib Investigator’s Brochure, Version 09, GlaxoSmithKline Document Number RM2003/00513/06].

3.3.3. Ongoing Rat and Mouse, 2-Year (“Lifetime”) Carcinogenicity Studies

Darapladib was given by oral gavage to male and female rodents starting from before sexual maturation and continuing every day throughout their lifetime for up to 2 years. Overall, the data suggest drug-related increases in the incidence of adenomas and/or adenocarcinomas of the jejunum in male mice and male rats given higher doses of darapladib. Specifically, drug exposure levels where these tumors were observed were 25-times greater in the male mice and 6.5-times greater in the male rats compared with drug exposures in humans at the clinical QD dose of 160 mg being tested in clinical trials. Tumors were not increased at a lower dose of darapladib in the rodent studies

where the blood levels were 7-times (mice) and 4-times (rats) higher than the blood levels of darapladib in humans at the 160 mg dose.

The relevance to humans of these rodent jejunal tumors is unknown; however, it is noted that:

- The incidences of jejunal tumors were less than 10% and only reached statistical significance in mice at the highest dose, indicating a weak signal.
- There is no pattern to suggest accelerated tumor latency.
- There was minimal evidence of tumor multiplicity within an animal.
- Darapladib was tested for and did not show evidence of genetic toxicity (DNA damage, mutations).

The Independent Data Safety and Monitoring Committee (IDMC) for the Phase III studies has reviewed the findings from the ongoing 2-year oral carcinogenicity studies in rodents and the updated safety information from the ongoing Phase III Program in June 2012. In addition, the IDMC met in August 2012 as part of the regularly scheduled reviews. The recommendations from the IDMC were to continue the studies without modifications. Finally, in December 2012, the IDMC Chairman reviewed data from both studies as part of the routine quarterly Chairman's reviews and recommended to continue the studies without modifications.

The exposure to darapladib is brief (28 days), therefore the risk of carcinogenicity is considered minimal.

For additional information refer to the Investigator's Brochure [Darapladib Investigator's Brochure, Version 09, GlaxoSmithKline Document Number RM2003/00513/06].

Table 1 Summary of Key Issues, Their Impact and Strategy to Mitigate Risk

Potential risk	Summary of data	Impact-eligibility criteria	Strategy-monitoring/stopping criteria
4-fluorobenzyl chloride (4-FBCl)	4-FBCl is not a genotoxic carcinogen, but at very high exposures when in prolonged contact with the rodent non-glandular stomach it is an irritant that can lead to tumours via a non-genotoxic mechanism.	None	Subjects will be instructed that darapladib tablets must be swallowed whole and not chewed, to maintain the integrity of the enteric coat.<br><br>Subjects will be instructed to take the tablets after eating breakfast in order to raise the pH of the stomach and provide additional protection in the rare event that drug is released in the stomach.
Theoretical concern regarding PAF accumulation with Lp-PLA_2 inhibitors	Inhibition of Lp-PLA_2 may have the potential to increase PAF concentrations in plasma which is positively correlated with anaphylaxis severity in Subjects with acute allergic reactions	Subjects with a history of anaphylaxis anaphylactoid reactions or severe allergic responses are excluded.	AEs will be closely watched as defined in the Time and Events table (See Section 6.1).
Carcinogenicity studies	Drug-related increases in the incidence of adenomas and/or adenocarcinomas of the jejunum in male mice and male rats given higher doses of darapladib was observed in 2-year (“lifetime”) carcinogenicity rodent studies.	The exposure to darapladib in this study is brief (28 days), therefore the risk of carcinogenicity is considered minimal.	AEs will be closely watched as defined in the Time and Events table (See Section 6.1).

4. STUDY POPULATION

4.1. Number of Subjects

Approximately 24 Subjects will be enrolled such that approximately 18 Subjects could be evaluable for PK analysis.

4.2. Eligibility Criteria

Specific information regarding warnings, precautions, contraindications, AEs, and other pertinent information on the GSK investigational product or other study treatment that may impact Subject eligibility is provided in the IB supplement(s), product label.

Deviations from inclusion and exclusion criteria are not allowed because they can potentially jeopardize the scientific integrity of the study, regulatory acceptability or Subject safety. Therefore, adherence to the criteria as specified in the protocol is essential.

4.2.1. Inclusion Criteria

A Subject will be eligible for inclusion in this study only if all of the following criteria apply:

1. Healthy Chinese males or females aged between 18 and 45 years of age inclusive, at the time of signing the informed consent.
    - Healthy as determined by a responsible and experienced physician, based on a medical evaluation including medical history, physical examination, laboratory tests and cardiac monitoring. A Subject with a clinical abnormality or laboratory parameters which is/are not specifically listed in the inclusion or exclusion criteria, outside the reference range for the population being studied may be included only if the Investigator (in consultation with the GSK Medical Monitor if required) agree and document that the finding is unlikely to introduce additional risk factors and will not interfere with the study procedures.
2. Of Chinese origin – defined as being born in mainland China, having four ethnic Chinese grandparents.
3. Body weight ≥50 kg and BMI within the range 19 to 24 kg/m^2 (inclusive).
4. A female Subject is eligible to participate if she is of:
    - Non-childbearing potential defined as pre-menopausal females with a documented tubal ligation or hysterectomy [for this definition, “documented” refers to the outcome of the investigator's/designee’s review of the Subject's medical history for study eligibility, as obtained via a verbal interview with the Subject or from the Subject’s medical records]; or postmenopausal defined as 12 months of spontaneous amenorrhea [in questionable cases a blood sample with simultaneous follicle stimulating hormone (FSH) >40 MlU/ml and estradiol < 40 pg/ml (<147 pmol/L) is confirmatory].

- Child-bearing potential with negative pregnancy test as determined by blood human chorionic gonadotropin (hCG) test at screening , urine hCG test prior to dosing (day -1) and
- Agrees to use 1 of the contraception methods listed in Section 4.3.1 from the time of Screening to sufficiently minimize the risk of pregnancy at that point. Female Subjects must agree to use contraception until the follow-up contact.

5. Capable of giving written informed consent, which includes compliance with the requirements and restrictions listed in the consent form
6. Alanine aminotransferase (ALT), alkaline phosphatise (ALP) and bilirubin ≤1.5x upper limit of normal (ULN) (isolated bilirubin >1.5xULN is acceptable if bilirubin is fractionated and direct bilirubin <35%).
7. Based on single or averaged QTc values of triplicate ECGs obtained over a brief recording period:
   - QT duration corrected for heart rate by Bazett's formula (QTcB) or QT duration corrected for heart rate by Fridericia's formula (QTcF) <450 msec;
   - QTc <480 msec in Subjects with Bundle Branch Block.

4.2.2. Exclusion Criteria

A Subject will not be eligible for inclusion in this study if any of the following criteria apply:

4.2.2.1. Criteria Based Upon Medical Histories

1. Current or chronic history of liver disease, or known hepatic or biliary abnormalities (with the exception of Gilbert's syndrome or asymptomatic gallstones).
2. History of regular alcohol consumption within 6 months of the study defined as: an average weekly intake of >14 drinks for males or >7 drinks for females. One drink is equivalent to 12 g of alcohol: 12 ounces (360 ml) of beer, 5 ounces (150 ml) of wine or 1.5 ounces (45 ml) of 80 proof distilled spirits.
3. Consumption of grapefruit or grapefruit juice within 7 days prior to first dose of study medication.
4. History of sensitivity to heparin or heparin-induced thrombocytopenia.
5. History of asthma, anaphylaxis or anaphylactoid reactions, severe allergic responses.
6. History of sensitivity to any of the study medications, or components thereof or a history of drug or other allergy that, in the opinion of the investigator or GSK Medical Monitor, contraindicates their participation.
7. Unable to refrain from the use of prescription or non-prescription drugs, including vitamins, herbal and dietary supplements (including St John's Wort) within 7 days (or 14 days if the drug is a potential enzyme inducer) or 5 half-lives (whichever is longer) prior to the first dose of study medication.

4.2.2.2. Criteria Based Upon Diagnostic Assessments

8. A positive test for Hepatitis B surface antigen (HBsAg) or positive Hepatitis C antibody result, or positive test for human immunodeficiency virus (HIV) antibody or Syphilis antibody at Screening.
9. A positive pre-study drug/alcohol screen.
10. Pregnant females as determined by positive blood hCG test at screening or urine hCG test prior to dosing (day -1).
11. A chest X-ray or computed tomography (CT) scan that reveals evidence of clinical significant abnormalities eg, tuberculosis. A chest X-ray must be taken at Day-1 if a chest X-ray or CT scan is not available within 6 months prior to that day.

4.2.2.3. Other Criteria

12. Where participation in the study would result in donation of blood or blood products in excess of 500 mL within a 56 day period.
13. Lactating females.
14. The Subject has participated in a clinical trial and has received an investigational product within the following time period prior to the first dosing day in the current study: 30 days, 5 half-lives or twice the duration of the biological effect of the investigational product (whichever is longer).
15. Exposure to more than 4 new chemical entities within 12 months prior to the first dosing day.
16. Unwillingness or inability to follow the procedures outlined in the protocol.

4.3. Lifestyle and/or Dietary Restrictions

4.3.1. Contraception Requirements

4.3.1.1. Female Subjects

Female Subjects of childbearing potential must not become pregnant and so must be sexually inactive by abstinence or use contraceptive methods with a failure rate of <1%. Female Subjects of childbearing potential with same sex partners (when this is their preferred and usual lifestyle) are not required to be abstinent or to use contraception.

Abstinence

Sexual inactivity by abstinence must be consistent with the preferred and usual lifestyle of the Subject. Periodic abstinence (e.g. calendar, ovulation, symptothermal, post-ovulation methods) and withdrawal are not acceptable methods of contraception.

Contraceptive Methods with a Failure Rate of <1%

- Non-hormonal intrauterine device or intrauterine system that meets the <1% failure rate as stated in the product label
- **Documented** male partner sterilization prior to **the female Subject's entry** into the study, and this male is the sole partner for that Subject. For this definition, "documented" refers to the outcome of the investigator's/designee's review of the Subject's medical history for study eligibility, as obtained via a verbal interview with the Subject or from the Subject's medical records.
- Male condom combined with a female diaphragm, either with or without a vaginal spermicide (e.g. foam, gel, cream or suppository).

NOTE: These allowed methods of contraception are only effective when used consistently, correctly and in accordance with the product label. The investigator is responsible for ensuring Subjects to understand how to properly use these methods of contraception.

4.3.2. Meals and Dietary Restrictions

- Subjects will not be allowed to drink grapefruit juice or eat grapefruit within 7 days prior to the first dose of study medication and until collection of the final blood sample in each session
- Water, soft drinks without caffeine or fruit juices (except grapefruit juice) may be consumed ad libitum beginning 4 hours after dosing (water may be consumed beginning 2 hours after dosing).
- Meals will be provided during the in-patient periods. A standard breakfast will be served approximately 1 hour before dosing of darapladib. Lunch and dinner will be served at approximately 4 and 10 hours after dosing respectively. An evening snack will be permitted up to 22:00 hours.
- A standard breakfast will be provided and given approximately 1 hour before dosing at each of the outpatient visits

4.3.3. Caffeine, Alcohol, and Tobacco

- During each dosing session, Subjects will abstain from ingesting caffeine- or xanthine-containing products (e.g. coffee, tea, cola drinks, chocolate) for 24 hours prior to the start of dosing until collection of the final PK and or PD sample during each session.
- During each dosing session, Subjects will abstain from alcohol for 24 hours prior to the start of dosing until collection of the final PK and or PD sample during each session.
- Subjects who use tobacco products will not be permitted while they are in the Clinical Unit.

4.3.4. Activity

Subjects will abstain from strenuous exercise for 48 hours prior to each blood collection for clinical laboratory tests. Subjects may participate in light recreational activities during studies (e.g., watch television, read).

4.4. Screen and Baseline Failures

Data for Screen and Baseline failures will be collected in source documentation at the site but will not be transmitted to GSK.

NOTE: Refer to Section 7.1.1 for details of the required assessments and report if a Subject meets the SAE criteria.

4.5. Withdrawal Criteria and Procedures

A Subject may withdraw from study treatment at any time at his/her own request, or may be withdrawn at any time at the discretion of the Investigator for safety, behavioral or administrative reasons.

Refer to Section 5.3 for dose adjustment/stopping criteria

Liver chemistry threshold stopping criteria have been designed to assure Subject safety and to evaluate liver event etiology (in alignment with the Food and Drug Administration (FDA) premarketing clinical liver safety guidance). See Section 5.3.1 for details.

4.6. Subject Completion

A completed Subject is 1 who has completed all phases of the study including the follow-up visit.

The end of the study is defined as the last Subject's last visit (LSLV).

5. STUDY TREATMENT

5.1. Investigational Product

	Study Treatment
**Product name:**	darapladib
**Dosage form:**	Enteric coated, free base (micronised) tablet
**Unit dose strength(s)/Dosage level(s):**	160 mg
**Route/ Administration/ Duration:**	Route: oral<br>Administered: daily<br>Duration: Single dose, followed by 28 days of repeat dosing
**Dosing instructions:**	Take 1 hour after breakfast, swallow whole, do not chew.
**Manufacturer/ source of procurement:**	GSK

5.2. Treatment Assignment

All Subjects will be assigned to receive 160 mg of EC micronised free-base darapladib as a single dose and as repeated daily doses for 28 days.

5.3. Subject Specific Dose Adjustment/Stopping Criteria

5.3.1. Liver Chemistry Stopping Criteria

Liver chemistry threshold stopping criteria have been designed to assure Subject safety and to evaluate liver event etiology (in alignment with the FDA premarketing clinical liver safety guidance).

Study treatment will be stopped for a Subject if the following liver chemistry stopping criteria is met:

- Alanine aminotransferase (ALT) ≥3xULN

NOTE: Refer to Appendix 1 for details of the required assessments if a Subject meets the above criteria**.**

5.3.2. QTc Withdrawal Criteria

A Subject that meets either criterion below will be withdrawn from the study. The QT correction formula used to determine discontinuation should be the same one used throughout the study.

- QTc>500 msec

If a Subject has underlying bundle branch block the following withdrawal criteria should be used instead:

Baseline QTc value (with underlying bundle branch	QTc withdrawal criteria
<450 msec	>500 msec
450-480 msec	≥530 msec

Withdrawal of Subjects is to be based on an average QTc value of triplicate ECGs. If an ECG demonstrates a prolonged QT interval, then obtain 2 more ECGs at different timepoints more than 5 minute apart after 15 minutes rest and then use the averaged QTc values of the 3 ECGs to determine whether the Subject should be discontinued from the study

5.3.3. Other Dose Adjustment/Stopping Safety Criteria

For an individual study participant, stopping criteria include, but are not limited to:

Severe signs or symptoms, or significant changes in any of the safety assessments, that put the safety of the individual at risk (e.g. ECG, vital signs, laboratory tests, etc), as judged by the Principal Investigator in consultation with the Medical Monitor if necessary.

5.4. Blinding

This will be an open-label study.

5.5. Packaging and Labeling

The contents of the label will be in accordance with all applicable regulatory requirements.

5.6. Preparation/Handling/Storage/Accountability

No special preparation of study treatment is required.

Study treatment must be dispensed or administered according to procedures described herein. Only Subjects enrolled in the study may receive study treatment. Only authorized site staff may supply or administer study treatment. All study treatment must be stored in a secure area with access limited to the Investigator and authorized site staff. Study treatment is to be stored up to 30°C, protected from light and moisture. Maintenance of a temperature log (manual or automated) is required.

The Investigator, institution, or the Head of the medical institution (where applicable) is responsible for study treatment accountability, reconciliation, and record maintenance. The Investigator or the Head of the medical institution (where applicable), or designated site staff (e.g., storage manager, where applicable) must maintain study treatment accountability records throughout the course of the study. The responsible persons will document the amount of study treatment received from and returned to GSK and the amount administered to Subjects. The required accountability unit for this study will be tablet. Discrepancies are to be reconciled or resolved. Unused investigational products

will be returned to and destroyed by GSK. Procedures for final disposition of unused study treatment are listed in the SPM.

Investigational product is not expected to pose significant occupational safety risk to site staff under normal conditions of use and administration. A Material Safety Data Sheet (MSDS)/equivalent document describing occupational hazards and recommended handling precautions either will be provided to the Investigator, where this is required by local laws, or is available upon request from GSK.

Precaution will be taken to avoid direct contact with the investigational product. A MSDS describing occupational hazards and recommended handling precautions will be provided to the Investigator.

5.7. Assessment of Compliance

When the individual dose for a Subject is prepared from a bulk supply, the preparation of the dose will be confirmed by a second member of the study site staff.

When Subjects are dosed at the study site, they will receive study treatment directly from the Investigator or designee, under medical supervision. The date and time of each dose administered in the clinic will be recorded in the source documents. The dose of study treatment and study participant identification will be confirmed at the time of dosing by a member of the study site staff other than the person administering the study treatment. Study site personnel will examine each Subject's mouth to ensure that the study treatment was ingested.

5.8. Treatment of Study Treatment Overdose

For this study, any dose of darapladib >160 mg within a 24 hour time period (± 1 hour) will be considered an overdose.

GSK does not recommend specific treatment for an overdose. The Investigator will use clinical judgment to treat any overdose.

5.9. Treatment After the End of the Study

Subjects will not receive any additional treatment from GSK after completion of the study because only healthy volunteers are eligible for study participation.

5.10. Concomitant Medications and Non-Drug Therapies

5.10.1. Permitted Medications

Paracetamol at doses of ≤2 grams/day is permitted for use any time during the study. Other concomitant medication may be considered on a case by case basis by the (Investigator in consultation with the GSK Medical Monitor [if required]).

5.10.2. Prohibited Medications and Non-Drug Therapies

Subjects must abstain from taking prescription or non-prescription drugs (including vitamins and dietary or herbal supplements), within 7 days (or 14 days if the drug is a potential enzyme inducer) or 5 half-lives (whichever is longer) prior to the first dose of study medication until completion of the Follow-up visit, unless in the opinion of the Investigator and Sponsor the medication will not interfere with the study.

6. STUDY ASSESSMENTS AND PROCEDURES

This section lists the procedures and parameters of each planned study assessment. The exact timing of each assessment is listed in the Time and Events Table Section 6.1. Whenever vital signs, 12-lead ECGs and blood draws are scheduled for the same nominal time, the assessments should occur in the following order: 12-lead ECG, vital signs, and blood draws.

Protocol waivers or exemptions are not allowed with the exception of immediate safety concerns. Therefore, adherence to the study design requirements, including those specified in the Time and Events Table, are essential and required for study conduct.

The timing and number of planned study assessments, including safety, PK, PD assessments may be altered during the course of the study based on newly available data (e.g. to obtain data closer to the time of peak plasma concentrations) to ensure appropriate monitoring. The change in timing or addition of time points for any planned study assessments must be approved and documented by GSK, but this will not constitute a protocol amendment. Any safety issues that require alteration of the safety monitoring scheme should be approved by the Institutional Review Board/Independent Ethics Committee (IRB/IEC). There will be 43 time points for PK blood sample collection and approximately 2 mL of blood will be collected each time. There will be 29 time points for PD blood sample collection and approximately 2 mL of blood will be collected each time. Haematological and chemical assessment will be tested for 5 times, 4 mL of blood will be collected for each test. Virological assessment will be tested for 1 time, 3 mL of blood will be collected. 2 ml of blood will be collected for blood pregnancy test at screening for female subjects. Approximately 180 mL of blood will be collected over the duration of the study. If Investigator consider that additional safety assessment is needed, extra blood sample may be required. Venous indwelling needle can be used due to frequently blood sampling in single dose session Day 1 and repeated dose session Day 28. The additional blood loss may be 10ml.

6.1. Time and Events Table

Single Dose Session

	**Screening** (up to 30 days prior to day -1)	**Day -1**	**Single dose session**																
**Day**			**Day 1**												**Day2**		**Day3**	**Day4**	**Day5**
**Time After Dose (Hours)**		**At admission**	**Pre dose** [1]	**0h**	**0.5h**	**1h**	**2h**	**3h**	**4h**	**6h**	**9h**	**10h**	**12h**	**15h**	**24h**	**36h**	**48h**	**72h**	**96h**
Informed consent	X																		
Admission to the Unit [2]		X																	
Inclusion/exclusion Criteria review	X	X																	
Demographic/Medical History Assessment	X																		
Full Physical Exam	X																		
Brief Physical Exam		X																	
Vital signs	X	X	X						X						X				X
12-lead ECG	X		X						X						X				X
Chest X Ray Exam		X																	
Hema/Chem/Urinalysis tests	X	X[6]													X				X
HIV, HBV, HCV, Syphilis screen	X																		
Urine drug/alcohol screen	X																		
Urine pregnancy test (women)		X																	
Blood hCG test (women)	X																		
Meal		X	X [3]						X			X			X	X			
Dosing				X															
Adverse Event Review [4]			X						X			X			X	X	X	X	X
PK Blood Samples			X		X	X	X	X	X	X	X		X	X	X	X	X	X	X
PD Blood Samples			X																
Concomitant Medication Review		X	X														X	X	X
Discharge [5]																	X		
Outpatient Visit	X																	X	X

1. All pre-dose procedures should be completed approximately 60 minutes before dosing
2. Subjects must be admitted in the morning of the day before dosing
3. A standard breakfast will be served approximately 1 hour before dosing of darapladib
4. AEs will NOT be recorded until after the first dose of study drug.
5. Subjects will be discharged after 48 hour post dose examinations
6. If Screening visit is within 14 days prior to Day -1, then Hema/Chem/Urinalysis tests at Day -1 can be exemption

Repeat Dose Session

Day:	RD 1	RD 2	RD 3	RD 4	RD 5	RD 6	RD 7	RD 8	RD 9	RD 10	RD 11	RD 12	RD 13	RD 14	RD 15	RD 16	RD 17	RD 18	RD 19	RD 20	RD 21	RD 22	RD 23	RD 24	RD 25	RD 26	RD 27	RD 28
Dosing	X	X	X	X	X	X	X	X	X	X	X	X	X	X	X	X	X	X	X	X	X	X	X	X	X	X	X	X
Inpatient stay																											X	X
Outpatient visit	X	X	X	X	X	X	X	X	X	X	X	X	X	X	X	X	X	X	X	X	X	X	X	X	X	X		
Vital signs														X														X
AE review	Ongoing																											
Concomitant Medication Review	Ongoing																											
Meal [1]	X	X	X	X	X	X	X	X	X	X	X	X	X	X	X	X	X	X	X	X	X	X	X	X	X	X	X	X
PK Blood Samples														X [2]												X [2]	X [2]	X [3]
PD Blood Samples														X [2]												X [2]	X [2]	X [3]

1. A standard breakfast will be provided and given approximately 1 hour before dosing at each of the outpatient visits.
2. Day 14, Day 26 and Day 27 of repeat dosing require trough (predose) PK and PD samples
3. Samples will be obtained at 0, 0.5, 1, 2, 3, 4, 6, 9, 12, 15, 24 hours post dose

PK/PD Sampling After Last Repeat Dose:

Day:	29	30	31	32	33	34	35	36	37	38	39	40	41	42	43	44	45	46	47	48	49	50	51	52	53	54	55	56	Follow-up
Outpatient visit			X	X	X	X	X			X			X			X			X			X			X			X	X
PK blood sample [1]	X	X	X	X	X	X	X			X			X			X			X			X			X			X	
PD blood sample [1]	X	X	X	X	X	X	X			X			X			X			X			X			X			X	
Discharge		X																											
12-lead ECG																													X
Vital signs																													X
Urine pregnancy test (women)																													X
Hema/Chem/Urinalysis tests																													X
AE review	X (Days 29–56)																												X
Concomitant Medication Review	X (Days 29–56)																												X

1. After last day of repeat dosing PK and PD samples will be obtained at 36, 48, 72, 96, 120, 144, 168, 240, 312, 384, 456, 528, 600 and 672 hours post-dose

6.2. Demographic/Medical History Assessments

The following demographic parameters will be captured: date of birth, gender, race and ethnicity.

Medical/medication/alcohol history will be assessed as related to the eligibility criteria listed in Section 4.2.

6.3. Safety

Planned timepoints for all safety assessments are listed in Section 6.1. Additional time points for safety tests (such as vital signs, physical exams and laboratory safety tests) may be added during the course of the study based on newly available data to ensure appropriate safety monitoring.

6.3.1. Physical Exams

- A complete physical examination will include assessments of the head, eyes, ears, nose, throat, skin, thyroid, neurological, lungs, cardiovascular, abdomen (liver and spleen), lymph nodes and extremities. Height and weight will also be measured and recorded.
- A brief physical examination will include assessments of the skin, lungs, cardiovascular system, and abdomen (liver and spleen).

6.3.2. Vital Signs

- Vital sign measurements to be measured in sitting position after 5 minutes rest will include systolic and diastolic blood pressure and pulse rate.

6.3.3. Electrocardiogram

- 12-lead ECGs will be obtained at designated timepoint during the study outlined in Section 6.1 using an ECG machine that automatically calculates the heart rate and measures PR, QRS, QT, and QTc intervals. Refer to Section 5.3.2 for QTc withdrawal criteria and additional QTc readings that may be necessary.

6.3.4. Clinical Laboratory Assessments

Hematology, clinical chemistry, urinalysis and additional parameters to be tested are listed below. Details for the preparation and shipment of samples will be provided by the local laboratory. Reference ranges for all safety parameters will be provided to the site by the laboratory.

If additional non-protocol specified laboratory assessments are performed at the site's local laboratory and result in a change in Subject management or are considered clinical significant by the Investigator (for example serious adverse events [SAE] or AE or dose modification) the results must be captured and sent to GSK along with other study data as defined in Section 11.2(Appendix 2).

Hematology, clinical chemistry, urinalysis and additional parameters to be tested are listed below:

Hematology

Platelet Count	*RBC Indices:*	*Automated WBC Differential:*
RBC Count	MCV	Neutrophils
WBC Count (absolute)	MCH	Lymphocytes
Reticulocyte Count	MCHC	Monocytes
Hemoglobin		Eosinophils
Hematocrit		Basophils

Clinical Chemistry

BUN	Potassium	AST (SGOT)	Total and direct bilirubin
Creatinine	Chloride	ALT (SGPT)	Uric Acid
Glucose, fasting	Total CO_2	GGT	Albumin
Sodium	Calcium	Alkaline phosphatase	Total Protein

NOTE: Details of Liver Chemistry Stopping Criteria and Follow-Up Procedures are given in Section 5.3.1

Routine Urinalysis

Specific gravity
pH, glucose, protein, blood and ketones by dipstick
Microscopic examination (if blood or protein is abnormal)

Other screening tests

HIV
Hepatitis B (HBsAg)
Hepatitis C (Hep C antibody)
Syphilis (Syphilis antibody)
FSH and estradiol (as needed in women of non-child bearing potential only)
Blood hCG at screening visit and Urine hCG at Day-1 (in females)
Alcohol and drug screen (to include at minimum: amphetamines, barbiturates, cocaine, opiates, cannabinoids and benzodiazepines).

6.4. Pharmacokinetics

6.4.1. Blood Sample Collection

Blood samples for PK analysis of Darapladib + SB-553253 will be collected at the time points indicated in Section 6.1. The actual date and time of each blood sample collection will be recorded. The timing of PK samples may be altered and/or PK samples may be obtained at additional time points to ensure thorough PK monitoring.

Blood samples (approximately 2 mL) for PK analysis will be collected into K_2 ethylenediamine tetraacetic acid (EDTA) tubes.

Processing, storage and shipping procedures are provided in the SPM.

6.4.2. Sample Analysis

Determination of Darapladib and SB-553253 plasma concentrations will be performed by Wuxi AppTec using a recently validated bioanalytical methodology. Raw data will be stored in the Good Laboratory Practices Archives, Wuxi AppTec. Plasma analysis will be performed by Bioanalytical Science and Toxicokinetics, drug metabolism and pharmacokinetics (DMPK), GSK.

6.5. Biomarkers/Pharmacodynamic Markers

6.5.1. Confirmed Biomarkers/Pharmacodynamic Markers

Atherosclerotic biomarker is selected as Lp-PLA_2 activity in this study. Blood samples for PD analysis of Lp-PLA_2 will be collected at the time points indicated in Section 6.1. The actual date and time of each blood sample collection will be recorded. Blood samples (approximately 2 mL) for PK analysis will be collected into ETDA vacutainer. PD sample will be sent to Quest Diagnostics Company and performed by the colorimetric activity method. Details of PD sample collection, processing, storage and shipment are provided in the SPM.

7. ADVERSE EVENTS , SERIOUS ADVERSE EVENTS , PREGNANCY

7.1. Adverse Events and Serious Adverse Events

The Investigator or site staff is responsible for detecting, documenting and reporting events that meet the definition of an AE or SAE.

7.1.1. Time period for collecting AE and SAE information

AEs will be collected from the start of study treatment and until the follow-up contact. Medical occurrences that begin prior to the start of study treatment but after obtaining informed consent may be recorded on the Medical History/Current Medical Conditions on case report form (CRF).

SAEs will be collected over the same time period as stated above for AEs. However, any SAEs assessed as related to study participation (e.g., protocol-mandated procedures, invasive tests, or change in existing therapy) or related to a GSK product will be recorded from the time a Subject consents to participate in the study up to and including any follow-up contact. All SAEs will be recorded and reported to GSK within 24 hours, as indicated in Section 11.2 (Appendix 2).

Investigators are not obligated to actively seek AEs or SAEs in former study participants. However, if the Investigator learns of any SAE, including a death, at any time after a Subject has been discharged from the study, and he/she considers the event reasonably related to the study treatment or study participation, the Investigator would promptly notify GSK.

NOTE: The method of detecting, recording, evaluating and follow-up of AEs and SAEs plus procedures for completing and transmitting SAE reports to GSK are provided in Section 11.2 (Appendix 2).

7.1.2. Definition of Adverse Events

An AE is any untoward medical occurrence in a patient or clinical investigation Subject, temporally associated with the use of a medicinal product, whether or not considered related to the medicinal product.

NOTE: An AE can therefore be any unfavorable and unintended sign (including an abnormal laboratory finding), symptom, or disease (new or exacerbated) temporally associated with the use of a medicinal product.

Events meeting the definition of an AE **include**:

- Any abnormal laboratory test results (hematology, clinical chemistry, or urinalysis) or other safety assessments (e.g., ECGs, radiological scans, vital signs measurements), including those that worsen from baseline, and felt to be clinically significant in the medical and scientific judgement of the Investigator.

- Exacerbation of a chronic or intermittent pre-existing condition including either an increase in frequency and/or intensity of the condition.
- New conditions detected or diagnosed after study treatment administration even though it may have been present prior to the start of the study.
- Signs, symptoms, or the clinical sequelae of a suspected interaction.
- Signs, symptoms, or the clinical sequelae of a suspected overdose of either study treatment or a concomitant medication (overdose per se will not be reported as an AE/SAE unless this is an intentional overdose taken with possible suicidal/self-harming intent. This should be reported regardless of sequelae).
- "Lack of efficacy" or "failure of expected pharmacological action" per se will not be reported as an AE or SAE. However, the signs and symptoms and/or clinical sequelae resulting from lack of efficacy will be reported if they fulfil the definition of an AE or SAE.
- The signs and symptoms and/or clinical sequelae resulting from lack of efficacy will be reported if they fulfil the definition of an AE or SAE. Also, "lack of efficacy" or "failure of expected pharmacological action" also constitutes an AE or SAE.

Events that **do not** meet the definition of an AE include:

- Any clinically significant abnormal laboratory findings or other abnormal safety assessments that are associated with the underlying disease, unless judged by the investigator to be more severe than expected for the Subject's condition.
- The disease/disorder being studied, or expected progression, signs, or symptoms of the disease/disorder being studied, unless more severe than expected for the Subject's condition.
- Medical or surgical procedure (e.g., endoscopy, appendectomy); the condition that leads to the procedure is an AE.
- Situations where an untoward medical occurrence did not occur (social and/or convenience admission to a hospital).
- Anticipated day-to-day fluctuations of pre-existing disease(s) or condition(s) present or detected at the start of the study that do not worsen.

7.1.3. Definition of Serious Adverse Events

If an event is not an AE per Section 7.1.2, then it cannot be an SAE even if serious conditions are met (e.g., hospitalization for signs/symptoms of the disease under study, death due to progression of disease, etc).

An SAE is any untoward medical occurrence that, at any dose:

a. Results in death

b. Is life-threatening

NOTE: The term 'life-threatening' in the definition of 'serious' refers to an event in which the Subject was at risk of death at the time of the event. It does not refer to an event, which hypothetically might have caused death, if it were more severe.

c. Requires hospitalization or prolongation of existing hospitalization

NOTE: In general, hospitalization signifies that the Subject has been detained (usually involving at least an overnight stay) at the hospital or emergency ward for observation and/or treatment that would not have been appropriate in the physician's office or out-patient setting. Complications that occur during hospitalization are AEs. If a complication prolongs hospitalization or fulfills any other serious criteria, the event is serious. When in doubt as to whether "hospitalization" occurred or was necessary, the AE should be considered serious.

Hospitalization for elective treatment of a pre-existing condition that did not worsen from baseline is not considered an AE.

d. Results in disability/incapacity, or

NOTE: The term disability means a substantial disruption of a person's ability to conduct normal life functions. This definition is not intended to include experiences of relatively minor medical significance such as uncomplicated headache, nausea, vomiting, diarrhea, influenza, and accidental trauma (e.g. sprained ankle) which may interfere or prevent everyday life functions but do not constitute a substantial disruption.

e. Is a congenital anomaly/birth defect

f. Medical or scientific judgment should be exercised in deciding whether reporting is appropriate in other situations, such as important medical events that may not be immediately life-threatening or result in death or hospitalization but may jeopardize the Subject or may require medical or surgical intervention to prevent 1 of the other outcomes listed in the above definition. These should also be considered serious. Examples of such events are invasive or malignant cancers, intensive treatment in an emergency room or at home for allergic bronchospasm, blood dyscrasias or convulsions that do not result in hospitalization, or development of drug dependency or drug abuse.

g. Is associated with liver injury and impaired liver function defined as:

- ALT ≥3xULN and total bilirubin* ≥2xULN (>35% direct), or
- ALT ≥3xULN and International Normalized Ratio （INR）** >1.5.

* Serum bilirubin fractionation should be performed if testing is available; if unavailable, measure urinary bilirubin via dipstick. If fractionation is unavailable and ALT ≥3xULN and total bilirubin ≥2xULN, then the event is still to be reported as an SAE.

** INR testing not required per protocol and the threshold value does not apply to Subjects receiving anticoagulants. If INR measurement is obtained, the value is to be recorded on the SAE form.

- Refer to Appendix 1 for the required liver chemistry follow-up instructions.

7.1.4. Prompt Reporting of SAEs to GSK

Once the Investigator determines that an event meets the protocol definition of an SAE, the SAE will be reported to GSK within 24 hours. Any follow-up information on a previously reported SAE will also be reported to GSK within 24 hours.

If the investigator does not have all information regarding an SAE, he/she will not wait to receive additional information before notifying GSK of the event and completing the appropriate data collection tool. The investigator will always provide an assessment of causality at the time of the initial report as described in Appendix 2.

7.1.5. Regulatory Reporting Requirements for SAEs

Prompt notification of SAEs by the investigator to GSK is essential so that legal obligations and ethical responsibilities towards the safety of Subjects are met.

GSK has a legal responsibility to notify both the local regulatory authority and other regulatory agencies about the safety of a product under clinical investigation. GSK will comply with country specific regulatory requirements relating to safety reporting to regulatory authorities, IRBs/IECs and investigators.

Investigator safety reports are prepared for suspected unexpected serious adverse reactions according to local regulatory requirements and GSK policy and are forwarded to Investigators as necessary. An Investigator who receives an Investigator safety report describing an SAEs or other specific safety information (e.g., summary or listing of SAEs) from GSK will file it with the IB and will notify the IRB/IEC, if appropriate according to local requirements.

7.2. Pregnancy

7.2.1. Time period for collecting pregnancy information

All pregnancies in female Subjects and/or female partners of male Subjects will be collected after the start of dosing and until Follow-up visit.

7.2.2. Action to be taken if pregnancy occurs

The Investigator will collect pregnancy information on any female Subject, who becomes pregnant while participating in this study. The Investigator will record pregnancy information on the appropriate form and submit it to GSK within 2 weeks of learning of a Subject's pregnancy. The Subject will also be followed to determine the outcome of the pregnancy. Information on the status of the mother and child will be forwarded to GSK. Generally, follow-up will be no longer than 6 to 8 weeks following the estimated delivery date. Any premature termination of the pregnancy will be reported.

While pregnancy itself is not considered to be an AE or SAE, any pregnancy complication or elective termination of a pregnancy for medical reasons will be recorded as an AE or SAE.

A spontaneous abortion is always considered to be an SAE and will be reported as such. Furthermore, any SAE occurring as a result of a post-study pregnancy and is considered reasonably related to the study treatment by the Investigator, will be reported to GSK as described in Section 7.1.4. While the Investigator is not obligated to actively seek this

information in former study participants, he or she may learn of an SAE through spontaneous reporting.

Any female Subject who becomes pregnant while participating will be withdrawn from the study.

7.2.3. Action to be taken if pregnancy occurs in a female partner of a male study Subject

The Investigator will attempt to collect pregnancy information on any female partner of a male study Subject who becomes pregnant while participating in this study. This applies only to Subjects who are randomized to receive study medication. After obtaining the necessary written informed consent from the female partner directly, the Investigator will record pregnancy information on the appropriate form and submit it to GSK within 2 weeks of learning of the male Subject's partner's pregnancy. The partner will also be followed to determine the outcome of the pregnancy. Information on the status of the mother and child will be forwarded to GSK. Generally, follow-up will be no longer than 6 to 8 weeks following the estimated delivery date. Any premature termination of the pregnancy will be reported.

8. DATA ANALYSIS AND STATISTICAL CONSIDERATIONS

8.1. Hypotheses and Treatment Comparisons

No formal hypothesis will be tested. An estimation approach will be taken and appropriate corresponding confidence intervals (CIs) will be used to address the comparisons of interest.

For the planned PK analysis, the PK parameters of darapladib (parent) and the pharmacologically active metabolite SB-553253 on Day 28 (**Repeat Dose Session**) will be compared to those on Day 1 (**Single Dose Session**) separately. The comparisons of interest will be expressed as the accumulation ratios (Ro, Rp, Rs and Rcmax), as the data permit, according to the equations below:

$$\text{Observed Accumulation Ratio } (R_o) = \frac{AUC_{(0-\tau)} \text{ of Day 28}}{AUC_{(0-\tau)} \text{ of Day 1}}$$

$$\text{Predicted Accumulation Ratio } (R_p) = \frac{AUC_{(0-\infty)} \text{ of Day 1}}{AUC_{(0-\tau)} \text{ of Day 1}}$$

$$\text{Steady-State Accumulation Ratio } (R_s) = \frac{AUC_{(0-\tau)} \text{ of Day 28}}{AUC_{(0-\infty)} \text{ of Day 1}}$$

$$\text{Cmax Accumulation Ratio } (R_{cmax}) = \frac{C_{max} \text{ of Day 28}}{C_{max} \text{ of Day 1}}$$

For metabolite, AUC and C_{max} metabolite to parent ratio will be calculated on each day (Day 1 Single Dose Session and Days 28 Repeat Dose Session).

For the planned PD analysis, percent inhibition of plasma Lp-PLA_2 activity will be calculated and summarized at each time point.

8.2. Sample Size Considerations

8.2.1. Sample Size Assumptions

Sample size is based on feasibility. The plan for this study was to enroll 24 Subjects to have 18 Subjects evaluable. However, some justification is provided below.

Based historical PK studies including LPL112498, LPL107988 and LPL110736, the CVw% for SB-480848 C_{max} ranges from 26.0% to 41.8%, and the CVw% for AUC ranges from 16.3% to 61.1% (see Table 2). With 18 Subjects evaluable for PK analysis, the half width of 90% CI for Rcmax will be within 22.0% of point estimate and for Ro, Rp and Rs will be within 16.6% of point estimate, assuming the CVw% to be observed in this study is 35.4% for C_{max} and 27.0% for AUC.

Table 2 CVw% for SB-480848

Study	CVw% for C_{max}	CVw% for AUC
LPL112498	41.8%	61.1%
LPL112498 (excluding potential outliers)	35.4%	27.0%
LPL107988	26.0%	18.3%.
LPL110736 (Japanese)	26.5%	16.3%

8.2.2. Sample Size Sensitivity

No sensitivity analysis was performed.

8.2.3. Sample Size Re-estimation

No sample size re-estimation will be performed.

8.3. Data Analysis Considerations

8.3.1. Interim Analysis

No formal interim analysis is planned. After the last dose of darapladib and before database freeze, Clinical Pharmacology Modeling and Simulation will assess preliminary PK data based on nominal time.

8.3.2. Final Analyses

The final planned analyses will be performed after all Subjects have completed the study and after database freeze.

8.3.2.1. Analysis Populations

Safety Population: All Subjects who receive at least 1 dose of darapladib will be included in the Safety Population. This population will be used in the evaluation of tolerability.

PK Population: All Subjects who receive at least 1 dose of darapladib and provide evaluable PK data will be included in the formal statistical analysis of the PK data.

PD Population: Subjects will be included in the summary statistics if they provide sufficient data to calculate percent inhibition of plasma Lp-PLA2 activity, i.e. provide data from baseline and at least 1 post-dose sample.

8.3.2.2. Safety Analyses

Safety data will be presented in tabular and/or graphical format and summarized descriptively according to GSK's Integrated Data Standards Library (IDSL) standards. Considering there is no placebo control group in the study, the observed safety data will

be compared to the safety results from other studies. Further details will be included in the Reporting and Analysis Plan (RAP).

8.3.2.3. Pharmacokinetic Analyses

PK analysis will be the responsibility of the Clinical Pharmacology Modeling & Simulation department within GSK. Plasma concentration time data for darapladib and SB-553253 will be analyzed by non-compartmental methods with WinNonlin [version 5.2 or higher]. Calculations will be based on the actual sampling times recorded during the study. From the plasma concentration-time data, the following PK parameters will be determined following both single dose and repeat dose sessions, as data permit: C_{max}, tmax, AUC, and t1/2. AUC and Cmax following single and repeat doses may be used for assessment of metabolite to parent ratio. Trough concentration ($C\tau$) samples collected on the specified days will be used to assess attainment of steady state, as appropriate. To estimate the extent of accumulation after repeat dosing, the Ro, the predicted accumulation ratio (Rp), the steady-state accumulation ratio (Rs) and Rcmax will be determined, as data permit.

PK data will be presented in graphical and/or tabular form and will be summarized descriptively. All PK data will be stored in the Archives, GSK Pharmaceuticals, R&D.

$C\tau$ data will be presented graphically for visual assessment of steady state.

Descriptive statistics (n, arithmetic mean and corresponding 95% CIs, standard deviation (SD), minimum, median, maximum, CVb%) will be calculated for metabolite to parent ratio by day.

Following $\log_e$-transformation, AUC and C_{max} will be separately analyzed using a mixed effect model, fitting day as fixed effects and Subject as a random effect. Point estimates and associated 90% CIs for the comparison of interests will be constructed using the residual variance. The point estimates and associated 90% CIs will then be exponentially back-transformed to provide point estimates and 90% CIs for the ratios (e.g., Ro, Rs and Rcmax).
Similar analysis will be done with Day 1 AUC ($0 - \infty$) and AUC ($0 - \tau$) data fitting PK parameter as fixed effect and Subject as random effect to provide point estimate and 90% CIs for the ratio of Rp.

The within-Subject coefficients of variation (CVw) for AUC and C_{max} will be calculated based on the $\log_e$–normal distribution.

Distributional assumptions underlying the statistical analyses will be assessed by visual inspection of residual plots. Normality will be examined by normal probability plots, while homogeneity of variance will be assessed by plotting the residuals against the predicted values for the model. Alternative analyses of the data will be performed if any of the model assumptions appear to be violated.

Descriptive statistics (n, arithmetic mean and corresponding 95% CIs, SD, minimum, median, maximum, CVb%) will be calculated for all PK endpoints by day.

In addition, for AUC (0-t), AUC (0 - ∞), AUC (0 - τ), Cmax, T½,and Cτ, geometric means and their 95% CIs will be calculated.

8.3.2.4. Pharmacodynamic/Biomarker Analyses

Lp-PLA_2 activity and its summary parameter, percent inhibition of plasma Lp-PLA_2 activity at each sampling point will be tabulated. Individual, mean and median of Lp-PLA_2 activity and percent inhibition of plasma Lp-PLA_2 activity-time figure will be produced.

Details of the planned statistical analyses will be provided in the RAP.

8.3.2.5. Pharmacokinetic/Pharmacodynamic Analyses

An exploratory analysis of the relationship between plasma concentrations of darapladib and plasma Lp-PLA_2 activity will be performed. Analysis of western PK/PD data has indicated a direct concentration-effect relationship between darapladib plasma concentrations and inhibition of Lp-PLA_2 activity that is characterised by a sigmoidal inhibitory E_{max} model. A similar exploratory analysis of the relationship between plasma concentrations of darapladib and plasma Lp-PLA_2 activity in Chinese Subjects will be performed for this study. Analyses will include appropriate graphical assessments of dose-response and concentration-response and model based (e.g. E_{max}) assessments. .Detailed PK/PD analysis will be specified in RAP.

9. STUDY GOVERNANCE CONSIDERATIONS

9.1. Posting of Information on Publicly Available Clinical Trial Registers

Study information from this protocol will be posted on publicly available clinical trial registers before enrollment of Subjects begins.

9.2. Regulatory and Ethical Considerations, Including the Informed Consent Process

Prior to initiation of a study site, GSK will obtain favourable opinion/approval from the appropriate regulatory agency to conduct the study in accordance with ICH GCP and applicable country-specific regulatory requirements.

The study will be conducted in accordance with all applicable regulatory requirements.

The study will also be conducted in accordance with ICH GCP, all applicable Subject privacy requirements, and, the guiding principles of the 2008 Declaration of Helsinki. This includes, but is not limited to, the following:

- IRB/IEC review and favorable opinion/approval to conduct the study and of any subsequent relevant amended documents
- Written informed consent (and any amendments) to be obtained for each Subject before participation in the study
- Investigator reporting requirements (e.g. reporting of AEs/SAEs/protocol deviations to IRB/IEC)

Written informed consent must be obtained from each Subject prior to participation in the study.

9.3. Quality Control (Study Monitoring)

In accordance with applicable regulations including GCP, and GSK procedures, GSK Monitors will contact the site prior to the start of the study to review with the site staff the protocol, study requirements, and their responsibilities to satisfy regulatory, ethical, and GSK requirements. When reviewing data collection procedures, the discussion will also include identification, agreement and documentation of data items for which the CRF will serve as the source document.

GSK will monitor the study and site activity to verify that the:

- Data are authentic, accurate, and complete.
- Safety and rights of Subjects are being protected.
- Study is conducted in accordance with the currently approved protocol and any other study agreements, GCP, and all applicable regulatory requirements.

The Investigator and the Head of the medical institution (where applicable) agrees to allow the Monitor direct access to all relevant documents

9.4. Quality Assurance

To ensure compliance with GCP and all applicable regulatory requirements, GSK may conduct a quality assurance assessment and/or audit of the site records, and the regulatory agencies may conduct a regulatory inspection at any time during or after completion of the study. In the event of an assessment, audit or inspection, the Investigator (and institution) must agree to grant the Advisor(s), Auditor(s) and Inspector(s) direct access to all relevant documents and to allocate their time and the time of their staff to discuss the conduct of the study, any findings/relevant issues and to implement any corrective and/or preventative actions to address any findings/issues identified.

9.5. Study and Site Closure

Upon completion or premature discontinuation of the study, the Monitor will conduct site closure activities with the Investigator or site staff, as appropriate, in accordance with applicable regulations including GCP, and GSK procedures.

In addition, GSK reserves the right to temporarily suspend or prematurely discontinue this study at any time for reasons including, but not limited to, safety or ethical issues or severe non-compliance. For multicenter studies, this can occur at 1 or more or at all sites. If GSK determines such action is needed, GSK will discuss this with the Investigator or the Head of the medical institution (where applicable), including the reasons for taking such action. When feasible, GSK will provide advance notification to the investigator or the Head of the medical institution, where applicable, of the impending action prior to it taking effect.

If the study is suspended or prematurely discontinued for safety reasons, GSK will promptly inform Investigators or the Head of the medical institution (where applicable) and the regulatory authorities of the suspension or premature discontinuation of the study and the reason(s) for the action. If required by applicable regulations, the Investigator or the Head of the medical institution (where applicable) must inform the IRB/IEC promptly and provide the reason for the suspension or premature discontinuation.

9.6. Records Retention

Following closure of the study, the Investigator or the Head of the medical institution (where applicable) must maintain all site study records, except for those required by local regulations to be maintained by someone else, in a safe and secure location. The records must be maintained to allow easy and timely retrieval, when needed (e.g., audit or inspection), and, whenever feasible, to allow any subsequent review of data in conjunction with assessment of the facility, supporting systems, and staff. Where permitted by local laws/regulations or institutional policy, some or all of these records can be maintained in a format other than hard copy (e.g., microfiche, scanned, electronic); however, caution needs to be exercised before such action is taken. The Investigator must assure that all reproductions are legible and are a true and accurate copy of the original, and meet accessibility and retrieval standards, including

re-generating a hard copy, if required. Furthermore, the Investigator must ensure there is an acceptable back-up of these reproductions and that an acceptable quality control process exists for making these reproductions.

GSK will inform the Investigator of the time period for retaining these records to comply with all applicable regulatory requirements. The minimum retention time will meet the strictest standard applicable to that site for the study, as dictated by any institutional requirements or local laws or regulations, or GSK standards/procedures; otherwise, the retention period will default to 5 years.

The Investigator must notify GSK of any changes in the archival arrangements, including, but not limited to, archival at an off-site facility or transfer of ownership of the records in the event the Investigator leaves the site.

9.7. Provision of Study Results to Investigators, Posting of Information on Publically Available Clinical Trials Registers and Publication

Where required by applicable regulatory requirements, an Investigator signatory will be identified for the approval of the clinical study report. The Investigator will be provided reasonable access to statistical tables, figures, and relevant reports and will have the opportunity to review the complete study results at a GSK site or other mutually-agreeable location.

GSK will also provide the Investigator with the full summary of the study results. The Investigator is encouraged to share the summary results with the study Subjects, as appropriate.

GSK will provide the Investigator with the randomization codes for their site only after completion of the full statistical analysis.

The results summary will be posted to the Clinical Study Register at the time of the first regulatory approval or within 12 months of any decision to terminate development. In addition, a manuscript will be submitted to a peer reviewed journal for publication no later than 12 months after the first approval or any decision to terminate development. When manuscript publication in a peer reviewed journal is not feasible, further study information will be posted to the GSK Clinical Study Register to supplement the results summary.

The results summary for an approved GSK medicinal product will be posted to the Clinical Study Register no later than 12 months after the LSLV or sooner if required by legal agreement, local law or regulation. In addition, a manuscript will be submitted to a peer-reviewed journal for publication within 18 months of LSLV. When manuscript publication in a peer reviewed journal is not feasible, further study information is posted to the GSK Clinical Study Register to supplement the results summary.

A manuscript will be progressed for publication in the scientific literature if the results provide important scientific or medical knowledge.

10. REFERENCES

Darapladib Investigator's Brochure, Version 09, GlaxoSmithKline Document Number RM2003/00513/06. Report Date: 12-Dec-2012

Garza CA, Montori VM, McConnell JP, et.al. Association between lipoprotein-associated phospholipase A2 and cardiovascular disease: a systemic review. Mayo Clin Proc. 2007;82:159-165.

James LP, Letzig L, Simpson PM, et al. Pharmacokinetics of Acetaminophen-Adduct in Adults with Acetaminophen Overdose and Acute Liver Failure. Drug Metab Dispos 2009; 37:1779-1784.

Macphee CH, Moores, KE., Boyd, HF., et al. Lipoprotein associated phospholipase A2, platelet activating growth factor acetylhydrolase, generates two bioactive products during the oxidation of low density lipoprotein. Use of a novel inhibitor. Biochem. J. 1999;338:479-487.

Packard CJ, O'Reilly DJ., Caslake MJ., et al. Lipoprotein associated phospholipase A2 as an independent predictor of coronary heart disease. New Eng J Med. 2000;343:1148-1155.

Ross R. Atherosclerosis: an inflammatory disease. New Eng J Med. 1999;340:115-126.

Sampson HA, Munoz-Fulong A, Campbell RL, et al. Second symposium on the definition and management of anaphylaxis: Summary report-Second National Institute of Allergy and Infectious Disease/Food Allergy and Anaphylaxis Network symposium. J Allergy Clin Immunol. 2006;117:391-397.

Tew DG, Southan C., Rice SQJ., et al. Purification, properties, sequencing and cloning of a lipoprotein associated serine dependent phospholipase involved in the oxidative modification of low density lipoproteins. Arterioscler Throm Vasc Biol. 1996;16:591-599.

Vadas P, Gold M, Perelman B, et al. Platelet-activating factor, PAF acetylhydrolase, and severe anaphylaxis. N Engl J Med 2008;358:28-35.

11. APPENDICES

11.1. Appendix 1: Liver Safety Process

Scenario 1 Healthy Volunteer Studies

The procedures listed below are to be followed if a Subject meets the liver chemistry stopping criteria defined in Section 5.3.1:

- Immediately withdraw the Subject from study treatment
- Notify the GSK Medical Monitor within 24 hours of learning of the abnormality to confirm the Subject's study treatment cessation and follow-up.
- Complete the "Safety Follow-Up Procedures" listed below.
- Complete the liver event CRFs. If the event also meets the criteria of an SAE (see Section 7.1.3), the SAE data collection tool will be completed separately with the relevant details.
- Upon completion of the safety follow-up withdraw the Subject from the study unless further safety follow up is required or GSK Medical Governance approval of drug restart is granted (see below).
- Do not restart investigational product unless written approval is granted by GSK Medical Governance (see below), whereupon the Subject continues in the study after completion of the liver chemistry monitoring.
- Refer to the Flow chart for a visual presentation of the procedures listed below.

Safety Follow-Up Procedures for Subjects with ALT ≥3xULN:

- Monitor Subjects weekly until liver chemistries (ALT, AST, alkaline phosphatase, bilirubin) resolve, stabilize or return to within baseline values.

Safety Follow-Up Procedures for Subjects with ALT ≥3xULN and total bilirubin ≥2xULN (>35% direct bilirubin); or ALT ≥3xULN and INR[1] > 1.5:

- <u>This event is considered an SAE</u> (see Section 7.1.3). Serum bilirubin fractionation should be performed if testing is available. If fractionation is unavailable, urinary bilirubin is to be measured via dipstick (a measurement of direct bilirubin, which would suggest liver injury).
- Make every reasonable attempt to have Subjects return to the clinic within 24 hours for repeat liver chemistries, additional testing, and close monitoring (with specialist or hepatology consultation recommended).
- Monitor Subjects <u>twice weekly</u> until liver chemistries (ALT, AST, alkaline phosphatase, bilirubin) resolve, stabilize or return to within baseline values.

[1] INR testing not required per protocol and the threshold value does not apply to Subjects receiving anticoagulants.

In addition, for <u>all</u> Subjects with ALT ≥ 3xULN, every attempt must be made to also obtain the following:

- Viral hepatitis serology including:
  - Hepatitis A immunoglobulin M (IgM) antibody.
  - HBsAg and Hepatitis B Core Antibody (IgM).
  - Hepatitis C RNA.
  - Cytomegalovirus IgM antibody.
  - Epstein-Barr viral capsid antigen IgM antibody (or if unavailable, obtain heterophile antibody or monospot testing).
  - Hepatitis E IgM antibody.
- Blood sample for PK analysis, obtained within 24 hs of last dose. Record the date/time of the PK blood sample draw and the date/time of the last dose of study treatment prior to blood sample draw on the CRF. If the date or time of the last dose is unclear, provide the Subject's best approximation. If the date/time of the last dose can not be approximated <u>OR</u> a PK sample can not be collected in the time period indicated above, **do not obtain a PK sample**. Instructions for sample handling and shipping are included in the SPM.
- Serum creatine phosphokinase and lactate dehydrogenase.
- Fractionate bilirubin, if total bilirubin ≥ 2xULN.
- Assess eosinophilia
- Record the appearance or worsening of clinical symptoms of hepatitis (fatigue, nausea, vomiting, right upper quadrant pain or tenderness, fever, rash or eosinophilia) on the AE CRF.
- Record use of concomitant medications, acetaminophen, herbal remedies, other over the counter medications, or putative hepatotoxins on the Concomitant Medications CRF.
- Record alcohol use on the Liver Events section of the CRF.

The following are required for Subjects with ALT ≥ 3xULN **and** bilirubin ≥ 2xULN (>35% direct) but are optional for other abnormal liver chemistries:

- Anti-nuclear antibody, anti-smooth muscle antibody, and Type 1 anti-liver kidney microsomal antibodies.
- Serum acetaminophen adduct high performance liquid chromatography assay (quantifies potential acetaminophen contribution to liver injury in Subjects with definite or likely acetaminophen use in the preceding week [James, 2009]).
- Liver imaging (ultrasound, magnetic resonance, or computerized tomography) to evaluate liver disease.

- The Liver Imaging and/or Liver Biopsy CRFs are also to be completed if these tests are performed.

Refer to the diagram below for a visual presentation of the procedures listed above.

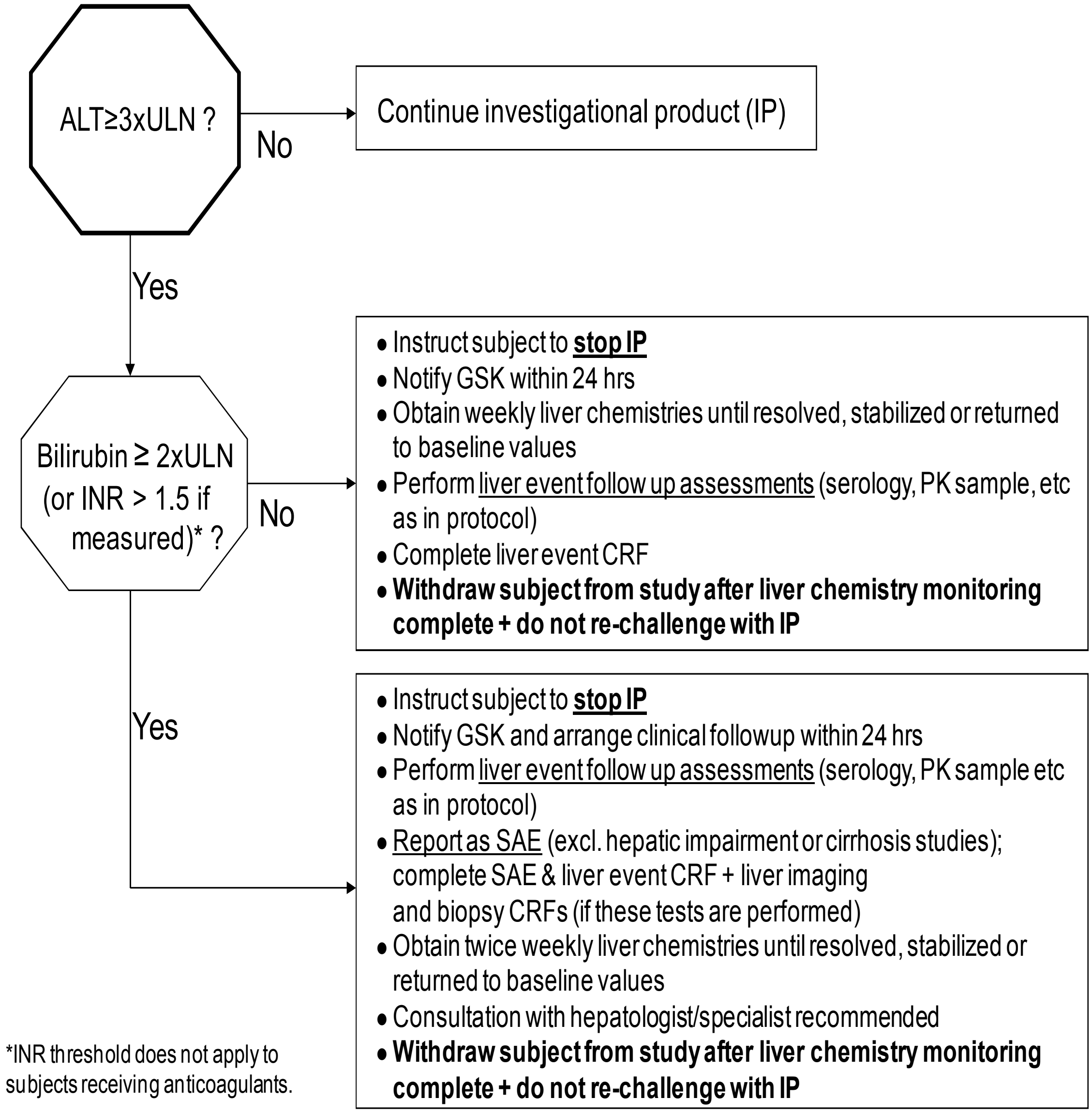

11.2. Appendix 2: Procedures for Detection, Evaluation, Follow-Up and Reporting of Adverse Events

Method of Detecting AEs and SAEs

Care will be taken not to introduce bias when detecting AEs and/or SAEs. Open-ended and non-leading verbal questioning of the Subject is the preferred method to inquire about AE occurrence. Appropriate questions include:

- "How are you feeling?"
- "Have you had any (other) medical problems since your last visit/contact?"
- "Have you taken any new medicines, other than those provided in this study, since your last visit/contact?"

Recording of AEs and SAEs

When an AE/SAE occurs, it is the responsibility of the Investigator to review all documentation (e.g., hospital progress notes, laboratory, and diagnostics reports) relative to the event. The Investigator will then record all relevant information regarding an AE/SAE in the appropriate data collection tool.

It is not acceptable for the Investigator to send photocopies of the Subject's medical records to GSK in lieu of completion of the GSK, AE/SAE data collection tool. However, there may be instances when copies of medical records for certain cases are requested by GSK. In this instance, all Subject identifiers, with the exception of the Subject number, will be blinded on the copies of the medical records prior to submission to GSK.

The Investigator will attempt to establish a diagnosis of the event based on signs, symptoms, and/or other clinical information. In such cases, the diagnosis will be documented as the AE/SAE and not the individual signs/symptoms.

Evaluating AEs and SAEs

Assessment of Intensity

The investigator will make an assessment of intensity for each AE and SAE reported during the study and will assign it to one of the following categories:

Mild: An event that is easily tolerated by the Subject, causing minimal discomfort and not interfering with everyday activities.

Moderate: An event that is sufficiently discomforting to interfere with normal everyday activities.

Severe: An event that prevents normal everyday activities.

An AE that is assessed as severe will not be confused with an SAE. Severity is a category utilized for rating the intensity of an event; and both AEs and SAEs can be assessed as severe. An event is defined as 'serious' when it meets at least one of the pre-defined outcomes as described in the definition of an SAE.

Assessment of Causality

The Investigator is obligated to assess the relationship between study treatment and the occurrence of each AE/SAE. A "reasonable possibility" is meant to convey that there are facts/evidence or arguments to suggest a causal relationship, rather than a relationship cannot be ruled out. The Investigator will use clinical judgment to determine the relationship. Alternative causes, such as natural history of the underlying diseases, concomitant therapy, other risk factors, and the temporal relationship of the event to the study treatment will be considered and investigated. The Investigator will also consult the Investigator Brochure (IB) and/or Product Information, for marketed products, in the determination of his/her assessment.

For each AE/SAE the Investigator must document in the medical notes that he/she has reviewed the AE/SAE and has provided an assessment of causality.

There may be situations when an SAE has occurred and the investigator has minimal information to include in the initial report to GSK. However, **it is very important that the Investigator always make an assessment of causality for every event prior to the initial transmission of the SAE data to GSK.** The Investigator may change his/her opinion of causality in light of follow-up information, amending the SAE data collection tool accordingly. The causality assessment is one of the criteria used when determining regulatory reporting requirements.

Follow-up of AEs and SAEs

After the initial AE/SAE report, the Investigator is required to proactively follow each Subject at subsequent visits/contacts. All AEs and SAEs will be followed until resolution, until the condition stabilizes, until the event is otherwise explained, or until the Subject is lost to follow-up.

The Investigator is obligated to perform or arrange for the conduct of supplemental measurements and/or evaluations as may be indicated or as requested by GSK to elucidate as fully as possible the nature and/or causality of the AE or SAE. The investigator is obligated to assist. This may include additional laboratory tests or investigations, histopathological examinations or consultation with other health care professionals. If a Subject dies during participation in the study or during a recognized follow-up period, the Investigator will provide GSK with a copy of any post-mortem findings, including histopathology.

New or updated information will be recorded in the originally completed data collection tool. The Investigator will submit any updated SAE data to GSK within the designated reporting time frames.

Reporting of SAEs to GSK

Once the investigator determines that an event meets the protocol definition of an SAE, the SAE will be reported to GSK **within 24 hours**. Any follow-up information on a previously reported SAE will also be reported to GSK within 24 hours.

If the investigator does not have all information regarding and SAE, he/she will not wait to receive additional information before notifying GSK of the event and completing the appropriate data collection tool. The investigator will always provide an assessment of causality at the time of the initial report.

After the study is completed at a given site, if the site receives a report of a new SAE from a study participant or receives updated data on a previously reported SAE, the site can report this information on a paper SAE form or to their GSK protocol contact by telephone.

GSK contacts for SAE receipt can be found at the beginning of this protocol on the Sponsor/Medical Monitor Contact Information page.

11.3. Appendix 3: Clinical Criteria for Diagnosing Anaphylaxis

Anaphylaxis is likely when any one of the following 3 criteria is fulfilled [Sampson, 2006].

1. Acute onset of an illness (minutes to several hours) with involvement of the skin, mucosal tissue, or both (eg, generalized hives, pruritus or flushing, swollen lips-tongue-uvula)

   AND AT LEAST ONE OF THE FOLLOWING

   a. Respiratory compromise (eg, dyspnea, wheeze-bronchospasm, stridor, reduced PEF, hypoxemia)

   b. Reduced BP or associated symptoms of end-organ dysfunction (eg, hypotonia [collapse], syncope, incontinence)

2. Two or more of the following that occur rapidly after exposure *to a likely allergen for that patient* (minutes to several hours):

   a. Involvement of the skin-mucosal tissue (eg, generalized hives, itch-flush, swollen lips-tongue-uvula)

   b. Respiratory compromise (eg, dyspnea, wheeze-bronchospasm, stridor, reduced PEF, hypoxemia)

   c. Reduced BP or associated symptoms of end-organ dysfunction (eg, hypotonia [collapse], syncope, incontinence)

   d. Persistent gastrointestinal symptoms (eg, crampy abdominal pain, vomiting)

3. Reduced BP after exposure to known allergen for that patient (minutes to several hours):

   a. Infants and children: low systolic BP (age specific) or greater than 30% decrease in systolic BP[1]

   b. Adults: systolic BP of less than 90 mm Hg or greater than 30% decrease from that person's baseline

1. PEF, Peak expiratory flow; BP, blood pressure.
1. Low systolic blood pressure for children is defined as less than 70 mmHg from 1 month to 1 year, less than (70 mmHg + [2 x age]) from 1 to 10 years, and less than 90 mmHg from 11 to 17 years.

11.4 Appendix 4: Protocol Amendment Changes

AMENDMENT 1

CHANGE 1

Page 4

SPONSOR/MEDICAL MONITOR INFORMATION PAGE

PREVIOUS TEXT

**Role**	Name	Day Time Phone Number	After-hours Phone/Cell/ Pager Number	Fax Number	GSK Address
Primary Medical Monitor	Julia Zhu	+86 (0)21 6159 0766	+86 186 2191 05972	+86 (0)21 6159 0708	GSK R&D China<br>No.1 building, 917 Halei Road<br>Zhangjiang Hi-tech Park,<br>Pudong

REVISED TEXT

**Role**	Name	Day Time Phone Number	After-hours Phone/Cell/ Pager Number	Fax Number	GSK Address
~~Primary Medical Monitor~~	~~Julia Zhu~~	~~+86 (0)21 6159 0766~~	~~+86 186 2191 05972~~	~~+86 (0)21 6159 0708~~	~~GSK R&D China~~<br>~~No.1 building, 917 Halei Road~~<br>~~Zhangjiang Hi-tech Park,~~<br>~~Pudong~~
Primary Medical Monitor	**Ray Tao**	**+86 (0)21 6159 0868**	**+86 139 1788 7601**	**+86 (0)21 6159 0705**	**GSK R&D China**<br>**No.1 building, 917 Halei Road Zhangjiang Hi-tech Park, Pudong**

Rationale: Medical monitor changed from Julia Zhu to Ray Tao

CHANGE 2

4.1 Number of Subjects

Page 20

PREVIOUS TEXT

Approximately 24 Subjects will be enrolled such that approximately 20 Subjects could be evaluable for PK analysis.

REVISED TEXT

Approximately 24 Subjects will be enrolled such that approximately ~~20~~**18** Subjects could be evaluable for PK analysis.

Rationale: Subjects number recalculate according to study feasibility.

CHANGE 3

Page 21

4.2.1. Inclusion Criteria

PREVIOUS TEXT

4. A female Subject is eligible to participate if she is of:

- Child-bearing potential with negative pregnancy test as determined by urine human chorionic gonadotropin (hCG) test at screening or prior to dosing and

REVISED TEXT

4. A female Subject is eligible to participate if she is of:

- Child-bearing potential with negative pregnancy test as determined by **blood** human chorionic gonadotropin (hCG) test at screening or **urine hCG test** prior to dosing **(day -1)** and

Rationale: According to PI's suggestion to clarify pregnancy test procedure and method, urine test with the exception of Screening Visit

CHANGE 4

Page 22

4.2.2.2. Criteria Based Upon Diagnostic Assessments

PREVIOUS TEXT

8. A positive test for Hepatitis B surface antigen (HBsAg) or positive Hepatitis C antibody result, or positive test for human immunodeficiency virus (HIV) antibody at Screening.

REVISED TEXT

8. A positive test for Hepatitis B surface antigen (HBsAg) or positive Hepatitis C antibody result, or positive test for human immunodeficiency virus (HIV) antibody or **syphilis antibody** at Screening.

Rationale: Add syphilis antibody examination according to medical monitor's suggestion to exclude Syphilis

CHANGE 5

Page 22

4.2.2.2 Criteria Based Upon Diagnostic Assessments

PREVIOUS TEXT

10. Pregnant females as determined by positive urine hCG test at screening or prior to dosing.

REVISED TEXT

Pregnant females as determined by positive **blood** hCG test at screening or **urine hCG test** prior to dosing **(day -1)**

Rationale: According to PI's suggestion to clarify pregnancy test procedure and method, urine test with the exception of Screening Visit

CHANGE 6

Page 22

4.2.2.2. Criteria Based Upon Diagnostic Assessments

PREVIOUS TEXT

…

REVISED TEXT

Add

11. **A chest X-ray or computed tomography (CT) scan that reveals evidence of clinical significant abnormalities. A chest X-ray must be taken at day -1 , if a chest X-ray or CT scan is not available within 6 months prior to that day.**

Rationale: According to PI's suggestion to exclude clinical significant abnormalities.

CHANGE 7

Page 24

4.4.Screen and Baseline Failures

PREVIOUS TEXT

Data for Screen and Baseline failures will be collected in source documentation at the site but will not be transmitted to GSK.

REVISED TEXT

Add

NOTE: Refer to Section 7.1.1 for details of the required assessments and report if a Subject meets the SAE criteria.

Rationale: To clarify the SAE assessments and report procedure when Screen and Baseline failure happens

CHANGE 8

Page 25

6.1 Investigational Product

PREVIOUS TEXT

Instructions for medical device use are provided in the study procedure manual (SPM).

REVISED TEXT

Delete

~~**Instructions for medical device use are provided in the study procedure manual (SPM).**~~

Rationale: Typo error

CHANGE 9

Page 29

6.1 Time and Events Table

Single Dose Session

PREVIOUS TEXT

	Screening (up to 30 days prior to day 1)	Single dose session																	
**Day**		**Day -1**	**Day 1**												**Day2**		**Day3**	**Day4**	**Day5**
**Time After Dose (Hours)**		**At admission**	**Pre dose [1]**	**0h**	**0.5h**	**1h**	**2h**	**3h**	**4h**	**6h**	**9h**	**10h**	**12h**	**18h**	**24h**	**32h**	**48h**	**72h**	**96h**
Informed consent	X																		
Admission to the Unit [2]		X																	
Demographic/Medical History Assessment	X																		
Full Physical Exam	X																		
Brief Physical Exam		X																	
Vital signs	X	X	X						X						X				X
12-lead ECG	X		X						X						X				X
Hema/Chem/Urinalysis tests	X	X													X				X
HIV, HBV, HCV screen	X																		
Urine drug/alcohol screen	X																		
Urine pregnancy test (women)	X	X																	
Meal		X	X [3]							X		X			X	X			
Dosing				X															
Adverse Event Review [4]			X						X			X			X	X	X	X	X
PK Blood Samples			X		X	X	X	X	X	X	X		X	X	X	X	X	X	X
PD Blood Samples			X																
Concomitant Medication Review		X	X														X	X	X
Discharge [5]																	X		
Outpatient Visit	X																	X	X

2.Subjects will be admitted in the morning of the day before dosing

REVISED TEXT

	**Screening** (up to 30 days prior to day <u>**-1**</u>)	<u>**Day -1**</u>	<u>**Single dose session**</u>																
**Day**			**Day 1**												<u>**Day2**</u>		**Day3**	**Day4**	**Day5**
**Time After Dose (Hours)**		**At admission**	**Pre dose** [1]	**0h**	**0.5h**	**1h**	**2h**	**3h**	**4h**	**6h**	**9h**	**10h**	**12h**	<u>**15h**</u>	**24h**	<u>**36h**</u>	**48h**	**72h**	**96h**
Informed consent	X																		
Admission to the Unit [2]		X																	
<u>**Inclusion/exclusion Criteria review**</u>	X	X																	
Demographic/Medical History Assessment	X																		
Full Physical Exam	X																		
Brief Physical Exam		X																	
Vital signs	X	X	X						X						X				X
12-lead ECG	X		X						X						X				X
<u>**Chest X Ray Exam**</u>		<u>**X**</u>																	
Hema/Chem/Urinalysis tests	X	X													X				X
HIV, HBV, HCV, <u>**Syphilis**</u> screen	X																		
Urine drug/alcohol screen	X																		
Urine pregnancy test (women)	~~X~~	X																	
<u>**Blood β-HCG test (women)**</u>	<u>**X**</u>																		
Meal		X	X [3]						<u>**X**</u>	~~X~~		X			X	X			
Dosing				X															
Adverse Event Review [4]			X						X			X			X	X	X	X	X
PK Blood Samples			X		X	X	X	X	X	X	X		X	X	X	X	X	X	X
PD Blood Samples			X																
Concomitant Medication Review		X	X														X	X	X
Discharge [5]																	X		
Outpatient Visit	X																	X	X

2. Subjects **<u>must</u>** be admitted in the morning of the day before dosing
6. **<u>If Screening visit is within 14 days prior to Day -1, then Hema/Chem/Urinalysis tests at Day -1 can be exemption</u>**

Rationale: Add Inclusion/exclusion Criteria review at screening and Day-1. Modify the examinations according the Inclusion and Exclusion criteria update. Change the PK blood sample and meal time according to study feasibility. It is mandatory for subjective to get admitted at the morning of Day -1. Specify the Hema/Chem/Urinalysis tests exemption at Day -1 to avoid duplication.

<u>CHANGE 10</u>

Page 31

6.1 Time and Events Table

Repeat Dose Session

PREVIOUS TEXT

3. Samples will be obtained at 0, 0.5, 1, 2, 3, 4, 6, 9, 12, 18, 24 hours post dose

REVISED TEXT

3.Samples will be obtained at 0, 0.5, 1, 2, 3, 4, 6, 9, 12, ~~18~~**<u>15,</u>** 24 hours post dose

Rationale: Change the PK blood sample time according to study feasibility.

<u>CHANGE 11</u>

Page 32

6.1 Time and Events Table

PK/PD Sampling After Last Repeat Dose

PREVIOUS TEXT

1. After last day of repeat dosing PK and PD samples will be obtained at 32, 48, 72, 96, 120, 144, 168, 240, 312, 384, 456, 528, 600 and 672 hours post-dose

REVISED TEXT

1.After last day of repeat dosing PK and PD samples will be obtained at ~~32~~**<u>36,</u>** 48, 72, 96, 120, 144, 168, 240, 312, 384, 456, 528, 600 and 672 hours post-dose

Rationale: Typo error change from 32 to 36.

<u>CHANGE 12</u>

Page 34

6.3.4. Clinical Laboratory Assessments

PREVIOUS TEXT

Other screening tests

HIV
Hepatitis B (HBsAg)
Hepatitis C (Hep C antibody)
FSH and estradiol (as needed in women of non-child bearing potential only)
Urine hCG (in females) at screening visit and Day-1
Alcohol and drug screen (to include at minimum: amphetamines, barbiturates, cocaine, opiates, cannabinoids and benzodiazepines).

REVISED TEXT

Other screening tests

HIV
Hepatitis B (HBsAg)
Hepatitis C (Hep C antibody)
**Syphilis (Syphilis antibody)**
FSH and estradiol (as needed in women of non-child bearing potential only)
**Blood hCG at screening visit and Urine hCG at Day-1 (in females)**
Alcohol and drug screen (to include at minimum: amphetamines, barbiturates, cocaine, opiates, cannabinoids and benzodiazepines).

Rationale: Add Syphilis antibody examination according medical monitor‘s suggestion to exclude Syphilis, and add blood hCG test at screening visit to exclude pregnant female subjects

CHANGE 13

Page 39

7. ADVERSE EVENTS , SERIOUS ADVERSE EVENTS , PREGNANCY AND MEDICAL DEVICES

PREVIOUS TEXT

7.1.4 Cardiovascular Events

Investigators will be required to fill out event specific data collection tools for the following AEs and SAEs:

- Myocardial infarction/unstable angina
- Congestive heart failure
- Arrhythmias
- Valvulopathy
- Pulmonary hypertension
- Cerebrovascular events/stroke and transient ischemic attack

- Peripheral arterial thrombosis
- Deep venous thrombosis
- Revascularization

This information should be recorded within 1 week of when the AE/SAE(s) are first reported.

7.1.5. Death Events

In addition, all deaths, whether or not they are considered SAEs, will require a specific death data collection tool to be completed. The death data collection tool includes questions regarding cardiovascular (including sudden cardiac death) and non cardiovascular death.

This information should be recorded within 1 week of when the death is first reported.

REVISED TEXT

7. ADVERSE EVENTS , SERIOUS ADVERSE EVENTS , PREGNANCY ~~AND MEDICAL DEVICES~~

7.1.4 Cardiovascular Events

~~Investigators will be required to fill out event specific data collection tools for the following AEs and SAEs:~~

- ~~Myocardial infarction/unstable angina~~
- ~~Congestive heart failure~~
- ~~Arrhythmias~~
- ~~Valvulopathy~~
- ~~Pulmonary hypertension~~
- ~~Cerebrovascular events/stroke and transient ischemic attack~~
- ~~Peripheral arterial thrombosis~~
- ~~Deep venous thrombosis~~
- ~~Revascularization~~

~~This information should be recorded within 1 week of when the AE/SAE(s) are first reported.~~

~~7.1.5. Death Events~~

~~In addition, all deaths, whether or not they are considered SAEs, will require a specific death data collection tool to be completed. The death data collection tool includes~~

~~questions regarding cardiovascular (including sudden cardiac death) and non cardiovascular death.~~

~~This information should be recorded within 1 week of when the death is first reported.Rationale: Delete Cardiovascular Event and Death Events collection according to new process of Cardiovascular Event & Death eCRF initiative~~

Rationale: Typo correction and delete Cardiovascular Event and Death Events collection according to new process of Cardiovascular Event & Death eCRF initiative document.

CHANGE 14

Page 41

8.2.1 Sample Size Assumptions

PREVIOUS TEXT

Sample size is based on feasibility. The plan for this study was to enroll 24 Subjects to have 20 Subjects evaluable. However, some justification is provided below.

Based historical PK studies including LPL112498, LPL107988 and LPL110736, the CVw% for SB-480848 C_{max} ranges from 26.0% to 41.8%, and the CVw% for AUC ranges from 16.3% to 61.1% (see Table 2). With 20 Subjects evaluable for PK analysis, the half width of 90% CI for Rcmax will be within 20.7% of point estimate and for Ro, Rp and Rs will be within 15.6% of point estimate, assuming the CVw% to be observed in this study is 35.4% for C_{max} and 27.0% for AUC.

REVISED TEXT

Sample size is based on feasibility. The plan for this study was to enroll approximately 24 Subjects to have ~~20~~**18** Subjects evaluable. However, some justification is provided below.

Based historical PK studies including LPL112498, LPL107988 and LPL110736, the CVw% for SB-480848 C_{max} ranges from 26.0% to 41.8%, and the CVw% for AUC ranges from 16.3% to 61.1% (see Table 2). With 20 Subjects evaluable for PK analysis, the half width of 90% CI for Rcmax will be within ~~20.7%~~**22.0%** of point estimate and for Ro, Rp and Rs will be within ~~15.6%~~ **16.6%** of point estimate, assuming the CVw% to be observed in this study is 35.4% for C_{max} and 27.0% for AUC.

Rationale: Subjects number recalculate according to study feasibility and statistician's recalculation

CHANGE 15

Page 52

APPENDIX 2: PROCEDURES FOR DETECTION, EVALUATION, FOLLOW-UP AND REPORTING OF ADVERSE EVENTS AND MEDICAL DEVICE INCIDENTS

PREVIOUS TEXT

PROCEDURES FOR DETECTION, EVALUATION, FOLLOW-UP AND REPORTING OF ADVERSE EVENTS AND MEDICAL DEVICE INCIDENTS

REVISED TEXT

PROCEDURES FOR DETECTION, EVALUATION, FOLLOW-UP AND REPORTING OF ADVERSE EVENTS ~~AND MEDICAL DEVICE INCIDENTS~~

Rationale: Typo error

CHANGE 16

Page 52

APPENDIX 2: PROCEDURES FOR DETECTION, EVALUATION, FOLLOW-UP AND REPORTING OF ADVERSE EVENTS AND MEDICAL DEVICE INCIDENTS

PREVIOUS TEXT

Subject-completed health outcomes questionnaires and the collection of AE data are independent components of the study. Responses to each question in the health outcomes questionnaire will be treated in accordance with standard scoring and statistical procedures detailed by the scale's developer. The use of a single question from a multidimensional health survey to designate a cause-effect relationship to an AE is inappropriate.

REVISED TEXT

~~Subject-completed health outcomes questionnaires and the collection of AE data are independent components of the study. Responses to each question in the health outcomes questionnaire will be treated in accordance with standard scoring and statistical procedures detailed by the scale's developer. The use of a single question from a multidimensional health survey to designate a cause-effect relationship to an AE is inappropriate.~~

Rationale: Clarify no health outcomes questionnaires including in this study.

AMENDMENT 2

CHANGE 1

Page 31

6.STUDY ASSESSMENTS AND PROCEDURES

PREVIOUS TEXT

The Institutional Review Board/Independent Ethics Committee (IRB/IEC) will be informed of any safety issues that require alteration of the safety monitoring scheme.

REVISED TEXT

Any safety issues that require alteration of the safety monitoring scheme **should be approved** by the Institutional Review Board/Independent Ethics Committee (IRB/IEC) .

Rationale:

Update the process according to EC review comments

CHANGE 2

Page 31

6.STUDY ASSESSMENTS AND PROCEDURES

PREVIOUS TEXT

Haematological and chemical assessment will be tested for 5 times and virological assessment will be tested for 1 time, 5 mL of blood will be collected for each test.

REVISED TEXT

Haematological and chemical assessment will be tested for 5 times, **4 mL** of blood will be collected for each test. Virological assessment will be tested for 1 time, **3 mL** of blood will be collected. 2 ml blood will be collected for blood pregnancy test at screening for female subjects.

Rationale:

Re-evaluate the blood sample volume according to investigator's suggestion.

CHANGE 3

Page 31

6. STUDY ASSESSMENTS AND PROCEDURES

PREVIOUS TEXT

…

REVISED TEXT

Add

Venous indwelling needle can be used due to frequently blood sampling in single dose session Day 1 and repeated dose session Day 28. The additional blood loss may be 10ml.

Rationale:

Add more information about potential blood sampling and additional blood loss according to investigator's suggestion.

CHANGE 4

Page 32

6.1 Time and Events Table

PREVIOUS TEXT

Blood β-HCG test (women)

REVISED TEXT

Blood **hCG** test (women)

Rationale:

Typo error
